# Functional Genomics and Comparative Lineage-Specific Region Analyses Reveal Novel Insights into Race Divergence in Verticillium dahliae

**DOI:** 10.1128/Spectrum.01118-21

**Published:** 2021-12-22

**Authors:** Dan Wang, Dan-Dan Zhang, Toshiyuki Usami, Lei Liu, Lin Yang, Jin-Qun Huang, Jian Song, Ran Li, Zhi-Qiang Kong, Jun-Jiao Li, Jun Wang, Steven J. Klosterman, Krishna V. Subbarao, Xiao-Feng Dai, Jie-Yin Chen

**Affiliations:** a State Key Laboratory for Biology of Plant Diseases and Insect Pests, Institute of Plant Protection, Chinese Academy of Agricultural Sciences, Beijing, China; b Institute of Vegetables and Flowers, Chinese Academy of Agricultural Sciences, Beijing, China; c Graduate School of Horticulture, Chiba University, Matsudo City, Japan; d Department of Plant Pathology, University of California, Davis, Salinas, California, USA; e United States Department of Agriculture, Agricultural Research Service, Crop Improvement and Protection Research Unit, Salinas, California, USA; The Ohio State University

**Keywords:** *Verticillium dahliae*, race, lineage-specific region, effector, virulence

## Abstract

Verticillium dahliae is a widespread soilborne fungus that causes Verticillium wilt on numerous economically important plant species. In tomato, until now, three races have been characterized based on the response of differential cultivars to V. dahliae, but the genetic basis of race divergence in V. dahliae remains undetermined. To investigate the genetic basis of race divergence, we sequenced the genomes of two race 2 strains and four race 3 strains for comparative analyses with two known race 1 genomes. The genetic basis of race divergence was described by the pathogenicity-related genes among the three races, orthologue analyses, and genomic structural variations. Global comparative genomics showed that chromosomal rearrangements are not the only source of race divergence and that race 3 should be split into two genotypes based on orthologue clustering. Lineage-specific regions (LSRs), frequently observed between genomes of the three races, encode several predicted secreted proteins that potentially function as suppressors of immunity triggered by known effectors. These likely contribute to the virulence of the three races. Two genes in particular that can act as markers for race 2 and race 3 (*VdR2e* and *VdR3e*, respectively) contribute to virulence on tomato, and the latter acts as an avirulence factor of race 3. We elucidated the genetic basis of race divergence through global comparative genomics and identified secreted proteins in LSRs that could potentially play critical roles in the differential virulence among the races in V. dahliae.

**IMPORTANCE** Deciphering the gene-for-gene relationships during host-pathogen interactions is the basis of modern plant resistance breeding. In the Verticillium dahliae-tomato pathosystem, two races (races 1 and 2) and their corresponding avirulence (*Avr*) genes have been identified, but strains that lack these two *Avr* genes exist in nature. In this system, race 3 has been described, but the corresponding *Avr* gene has not been identified. We *de novo*-sequenced genomes of six strains and identified secreted proteins within the lineage-specific regions (LSRs) distributed among the genomes of the three races that could potentially function as manipulators of host immunity. One of the LSR genes, *VdR3e*, was confirmed as the *Avr* gene for race 3. The results indicate that differences in transcriptional regulation may contribute to race differentiation. This is the first study to describe these differences and elucidate roles of secreted proteins in LSRs that play roles in race differentiation.

## INTRODUCTION

Verticillium dahliae is a widespread, soilborne fungus that causes vascular wilt on over 200 dicotyledonous plant species, resulting in billions of dollars in agricultural losses annually (1–3). At the conclusion of its infection cycle, V. dahliae forms microsclerotia, darkly pigmented resting structures (4), on senescent and dead host tissue. Microsclerotia can remain viable in the soil in the absence of hosts for at least 14 years (5) and can germinate repetitively (6) in response to host exudates and initiate new infections. The persistent nature of microsclerotia and its broad host range make V. dahliae very difficult to control. The use of resistant cultivars is an important strategy to manage Verticillium wilt because of the prolonged survival of microsclerotia following initial infestation (7).

Resistance or susceptibility of a host to a pathogen is broadly defined by the “gene-for-gene” theory (8), in which a single dominant host gene induces resistance following its recognition of the pathogen avirulence gene expression. Isolates that lack this avirulence gene or fail to express it escape host recognition and cause disease. Resistance in tomato against Verticillium wilt is governed by a single dominant locus called *Ve* (9). Soon after its identification, the *Ve* gene was incorporated into tomato cultivars, and all modern tomato cultivars carry this gene. Following the deployment of cultivars carrying the *Ve* gene in tomato production, V. dahliae strains that compromised the *Ve*-mediated resistance appeared within a few years (10–12), and these strains were designated race 2. Although sources of resistance to Verticillium wilt have been identified in several crops, including strawberry (13), sunflower (14), and mint (15), clear delineation of races based on the response of differential cultivars has only been achieved in tomato (10) and lettuce (16). Even though strains that could neither be characterized as race 1 or 2 existed, a method to assign a virulence phenotype for such isolates was not available. Thus, for more than 50 years, the V. dahliae populations were mainly composed of races 1 and 2 on tomato and lettuce (10–12, 17). A new source of resistance to race 2, identified in *Solanum neorickii* (18), was bred into rootstock cultivars such as Aibou and Ganbarune-Kari that served as differential cultivars, allowing the splitting of race 2 into races 2 and 3. Thus, V. dahliae infecting tomato can be split into three races. Resistance to race 2 in Aibou was modulated by a single gene designated V2 (18). Apart from *Ve1* from tomato, orthologues of *Ve1* have also been identified in many plant species, including lettuce (16, 19), cotton (20, 21), and Vitis vinifera (22). However, the presence of a corresponding race 2 resistance gene in other plant species has not been established.

With the advent of genomics and an innovative comparative population genomics approach, the avirulence factor in race 1 isolates, *Ave1*, was characterized in a subset of race 1 isolates (23). *Ave1* encodes a secreted cysteine-rich effector that confers avirulence in race 1 isolates of V. dahliae, which can induce cell death in tobacco expressing *Ve1* (23). In addition, *Ave1* was horizontally transferred to V. dahliae from a bacterial donor by an unknown mechanism and resides in a lineage-specific region (LSR) surrounded by transposable elements (24, 25). Similarly, comparative population genomics analyses have led to the identification of *Av2*, the gene encoding the avirulence factor in race 2, which also resides in a lineage-specific region that is enriched in repetitive elements (26). Furthermore, the presence of *Ave1* and *Av2* is not always mutually exclusive in V. dahliae isolates, since both avirulence factors can be encoded within a single strain (26).

Deciphering the genetic basis of important biological characteristics is at the heart of comparative genomics. Since the first V. dahliae genome was published (27), over 20 genomes from various V. dahliae strains have been released. Using these resources, the genetic basis of differentiating the defoliating and nondefoliating pathotypes was uncovered by comparing multiple genomes of both pathotypes of V. dahliae from cotton. Furthermore, the G-LSR2 present exclusively in the defoliating strains was horizontally transferred from the fungus Fusarium oxysporum f. sp. *vasinfectum*. The genes within the G-LSR-2 region encode orthologues for the biosynthesis of *N*-acylethanolamine 12:0, a compound that induces defoliation (28, 29). As important as the identification of the genetic basis underlying race divergence in V. dahliae (10–12, 17, 18) is, nearly all of the genomic studies thus far have focused on the genome comparison and identification of avirulence genes after the first race 1 genome was released (24, 26). Functional genomics to delineate the differences between races remains to be elucidated. Without the functional genomics approach to support comparative sequence analyses, a thorough understanding of the genetic basis of race divergence in V. dahliae is not possible.

We therefore sequenced the genomes of two race 2 strains and four race 3 strains that were characterized in previous studies (18) and conducted comparative functional genomics with two known race 1 genomes from tomato (JR2) (24) and lettuce (VdLs.16) (30). The objectives of this study were (i) to perform genomic analyses of pathogenicity-related genes among the three races, (ii) to analyze the evolution of races in V. dahliae by analyses of orthologues and genomic structural variations, (iii) to investigate the potential functions of lineage-specific regions among the three races, and (iv) to identify the potential effectors in the lineage-specific regions of races 2 and 3 strains and determine their roles in pathogenicity during the interactions with their host.

## RESULTS

### Characteristics of the sequenced race 2 and 3 genomes from tomato.

To understand the genomic basis of the V. dahliae nonrace 1 strains that had reportedly diverged into races 2 and 3, two race 2 strains (TO22 and Ud1-4-1) and four race 3 strains (HoMCLT, Gf-Cb5, GF1300, and GF1192) were sequenced (Table S1 in the supplemental material). Assemblies of the sequences from these strains revealed genome sizes in the 34.02- to 35.32-Mb range with the *N*_50_ length up to 3.5 Mb, and the proportional lengths of the top eight scaffolds relative to whole-genome length were in the range of 83.2 to 94.7% (Table 1; Fig. S1; Table S2 to S4). This indicated that the genome assemblies were of high quality when assuming the V. dahliae genome is composed of eight chromosomes (24, 27). Analysis of the genome features revealed that the ratio of total transposon sequence length was in the range of 7.62 to 10.70%; the two most common transposons were *Gypsy* and *Copia* across the different strains (Table S5). Gene prediction results and their analyses indicated that the sequenced genomes had similar gene densities and numbers of genes (Table 1). Interestingly, HoMCLT and GF1300, the two race 3 strains, present a different gene structure relative to the other sequenced genomes with shorter gene lengths (versus the JR2 genome, *P < *9.66E−37 and *P < *4.37E−36, respectively, based on unpaired Student’s *t* tests) and higher GC content (versus the JR2 genome, *P < *2.43E−19 and *P < *1.34E−20, respectively, based on unpaired Student’s *t* tests) (Table 1; Fig. S2). Although the analyses of genome/gene features revealed conservation as may be anticipated for an asexual fungus, gene length variation was notable, and the mechanism by which this occurs during V. dahliae evolution is unknown.

**TABLE 1:** Assembly statistics and features of the genome sequences of three races of Verticillium dahliae from tomato

Features	Race 2		Race 3				Race 1^*a*^	
	TO22	Ud1-4-1	HoMCLT	Gf-Cb5	GF1300	GF1192	VdLs.16	JR2
No. of scaffolds	14	15	12	12	11	14	15	8
Genome size (bp)	35,237,594	35,231,109	34,022,344	35,322,602	34,776,999	34,870,348	36,092,313	36,150,287
*N*_50_ length (Mb)	3.64	4.09	3.55	3.58	3.62	4.06	3.66	4.17
*N*_90_ length (Mb)	1.72	2.47	2.32	2.12	2.93	1.21	2.53	3.36
GC content (%)	53.84	53.88	54.52	53.62	53.77	53.76	53.80	53.88
Repeat rate (%)	10.21	10.70	7.62	10.45	9.89	9.87	10.75	11.22
Protein-coding genes	10,519	10,556	10,377	10,472	10,367	10,345	10,799	10,875
Gene density (genes Mb^−1^)	299	300	305	296	298	297	299	301
Mean gene length (bp)	1,751	1,746	1,533	1,743	1,536	1,750	1,747	1,752
GC content of genes (%)	58.63	58.63	59.42	58.66	59.42	58.69	58.56	58.49
Mean coding sequence length (bp)	1,531	1,532	1,408	1,529	1,410	1,536	1,530	1,537
GC content of coding sequence (%)	59.48	59.47	60.01	59.51	60.01	59.54	59.41	59.31
Mean exon per gene	2.92	2.88	2.38	2.89	2.38	2.89	2.89	2.87
Mean exon length (bp)	523	530	590	528	590	530	529	534
Mean intron per gene	1.93	1.89	1.39	1.90	1.39	1.90	1.89	1.88
Mean intron length (bp)	114	113	91	113	91	113	114	114
Intron GC %	52.37	52.25	52.37	52.24	52.38	52.26	52.26	52.25
Mean intergenic length (bp)	1,597	1,584	1,836	1,623	1,900	1,607	1,584	1,571

a GenBank assembly accession numbers for JR2 and VdLs.16 are SAMN02919271 and SAMN14604095, respectively.

### Expansion and contraction of the pathogenicity-related gene families among races of Verticillium dahliae.

**FIG 1:**
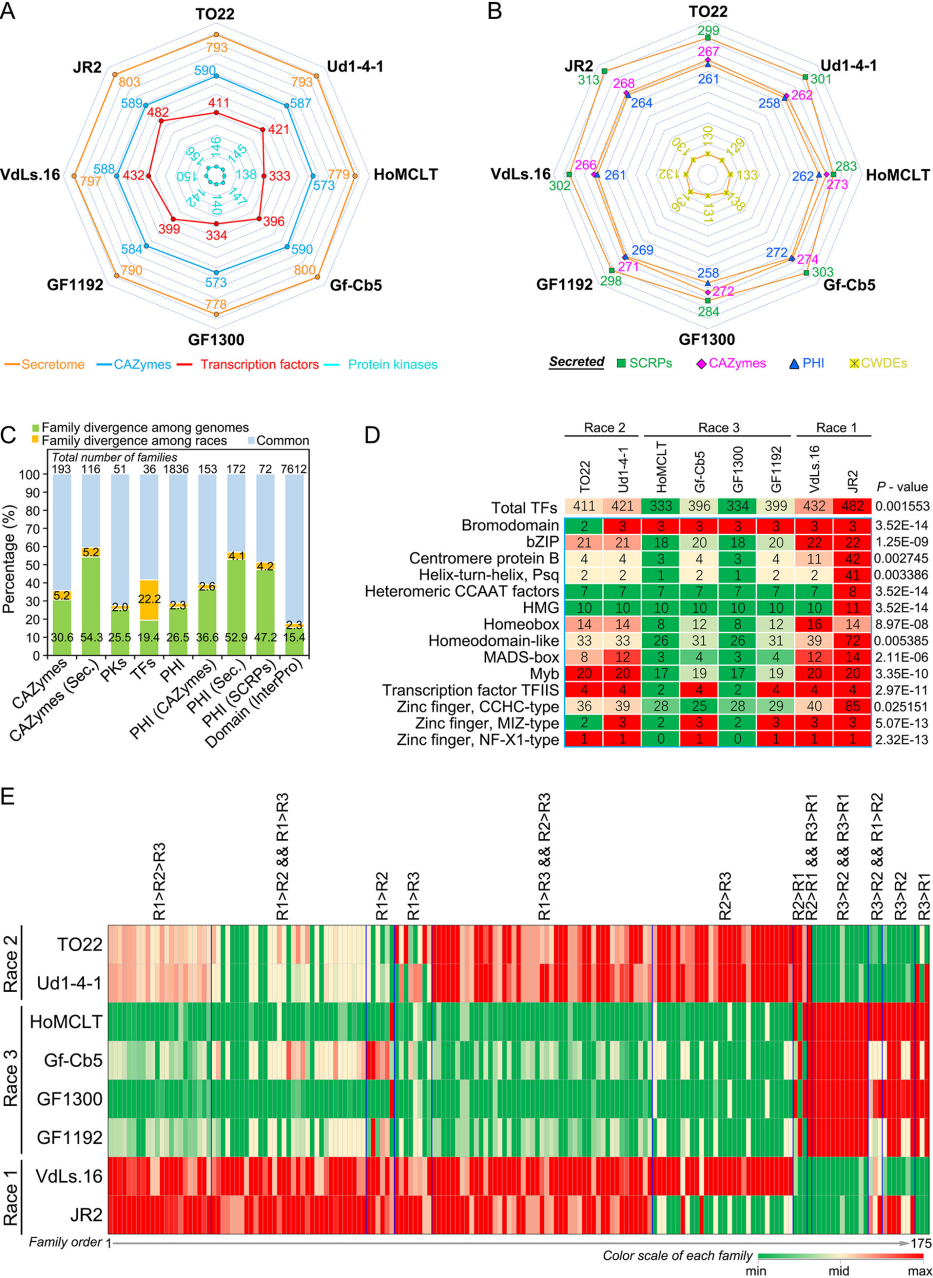
Functional analysis of the pathogenicity-related gene families among three races in Verticillium dahliae. (A) Comparison of total genes encoding pathogenicity-related proteins among the sequenced genomes, including those encoding secretory proteins, CAZymes, transcription factors, and protein kinases. JR2 and VdLs.16 are the two reference genomes. (B) Number of pathogenicity-related genes encoding secreted proteins among the sequenced genomes. (C) Divergence of the gene families among the sequenced genomes and three races. Gene families in common are represented by equal numbers of genes among the sequenced genomes; family divergence among genomes is represented by the number of genes of a certain race or from any two races that has expanded or contracted relative to the other races; PKs, protein kinases; TFs, transcription factors; PHI, pathogen-host interaction homologs; SCRPs, small cysteine-rich proteins; InterPro, the database used for conserved domain prediction; Sec., encodes protein with secretory characteristics. (D) Transcription factor divergence among three races of Verticillium dahliae. The significant divergence of each subfamily was detected by *F* test compared to the total number transcription factors among sequenced genomes. (E) Investigation of the functional divergence of predicted proteins by conserved domain (prediction by InterPro database) among the three races. R1, R2, and R3 represent race 1, race 2, and race 3, respectively; “&&” represents that both comparisons are true.

Comparison of the genomes of each of the races for gene content revealed notable conservation, as the gene numbers encoding secretory proteins, carbohydrate-active enzymes (CAZymes), and protein kinases (PKs) were similar in all three races (Fig. 1A and B; Table S6 to S10). However, investigations into these potential pathogenicity factors revealed various degrees of divergence among the genomes of the three races (Fig. 1C). Interestingly, the two race 3 genomes (HoMCLT and GF1300) encode significantly fewer transcription factors (TFs) than other races in comparison (Fig. 1A), resulting in 15 TF subfamily divergence among the three races (Fig. 1C and D; Table S11). This was especially pronounced in the JR2 strain that encodes 85 zinc finger CCHC-type proteins (Fig. 1D).

To further understand the genomic basis of race divergence in V. dahliae, the annotation of conserved domains was investigated using the InterPro database. There were 175 predicted, conserved domain catalogs that displayed divergence among the three races in V. dahliae, including regulatory factors, secondary metabolite biosynthesis genes, and functional enzymes (Fig. 1E; Table S13). Together, these results suggested that the genomes of the three races are highly conserved, but some functional divergence (especially the transcription regulators) that potentially contributes to the different biological characteristics among the three races in V. dahliae exists.

### Flexible orthologues among races in Verticillium dahliae.

**FIG 2:**
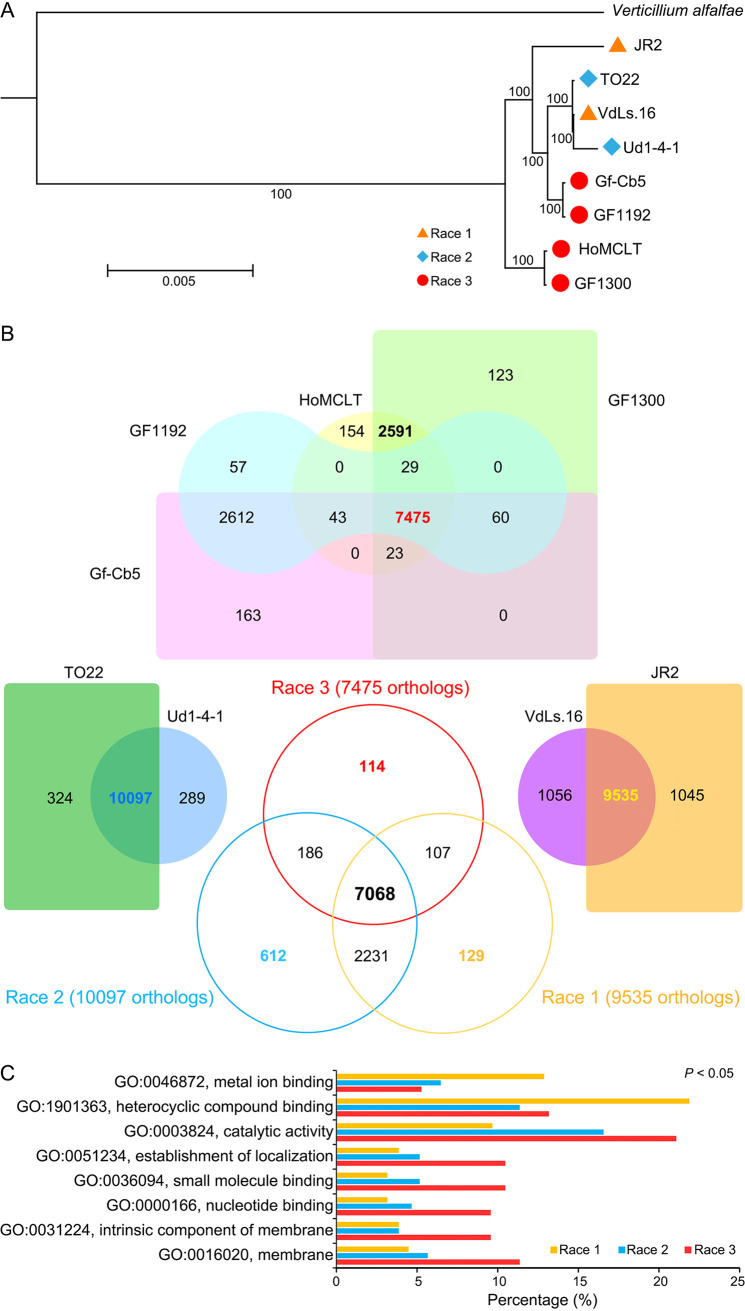
Orthologue clustering among races in Verticillium dahliae. (A) Phylogenetic tree of three races in V. dahliae. The phylogenetic tree was constructed using 6,238 concatenated single-copy orthologues using the maximum likelihood phylogeny with 1,000 bootstrap replicates. *Verticillium alfalfae* was used as an outgroup. (B) Investigation of orthologue clustering within and among race 2 in V. dahliae. Orthologues were clustered by OrthoMCL with both coverage and identities of >70%. The relationship of the orthologue among races is shown in a three-dimensional Venn diagram, with the common orthologues within race 3 (7,475 orthologues), race 2 (10,097 orthologues), and race 1 (9,535 orthologues), respectively. (C) GO annotation of the specific orthologues among three races in *Verticillium*. The significant clusters were selected by the Pearson chi-square test; *P < *0.05.

To further understand the race divergence in V. dahliae, the orthologue clustering of the sequenced and reference genomes was compared using the *Verticillium alfalfae* genome as the outgroup. Phylogenetic analysis using the 6,238 orthologues (single-copy genes) showed that the V. dahliae strains clustered into an independent branch with two separate subclades, but this alone could not explain the basis of race divergence as races 1 and 2 clustered into a subclade, with race 3 strains (HoMCLT and GF1300) forming a separate subclade (Fig. 2A). This suggested that the four sequenced race 3 strains diverged into two groups. Orthologue clustering showed that the majority of the genes clustered into common orthologues (>90%) between two strains each of races 1 and 2 (Fig. 2B). Unexpectedly, the orthologue clustering of the four race 3 strains displayed significant divergence with only 7,475 common orthologues in all race 3 strains, and the strains in the two divergent race 3 subclades clustered separately with 2,591 and 2,612 orthologues (Fig. 2A and B), respectively. The reduced orthologue clustering (gene pair: coverage of >30%; identities of >30%) revealed similar results in that the two race 3 groups yielded more than 1,000 specific orthologues (1,031 and 1,002 orthologues, respectively) (Fig. S3). These results suggested that the four sequenced race 3 strains diverged into two genotypes. Furthermore, clustering of the common orthologues showed that there are 114, 612, and 129 race-specific orthologues among races 3, 2, and 1, respectively (Fig. 2B; Table S14). Gene Ontology (GO) annotation showed that the known homologous genes also displayed divergence between race 1 and 3 strains, especially those involved in catalytic activity and binding function (Fig. 2C). In addition, investigations into pathogenicity-related genes revealed divergence among types of race-specific genes, including enrichment of predicted secreted proteins in race 3 and TFs in race 1 (Table S15). Together, these results further suggested significant divergence in the genomes of the three races as well as within race 3 strains, which potentially underlie a differential pathotype during tomato infection.

### The genome of race 3 diverged into two genotypes.

**FIG 3:**
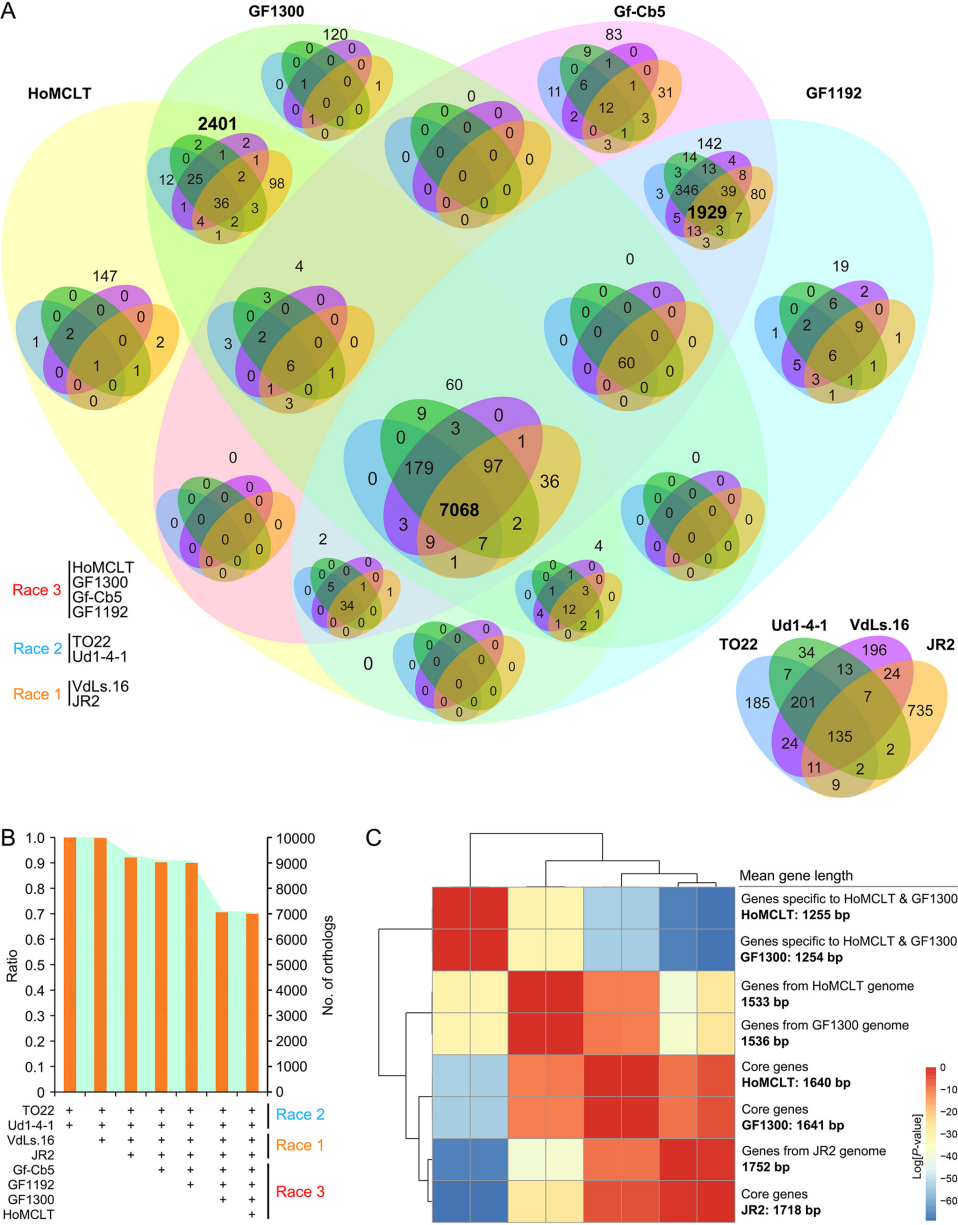
Orthologue divergence within the race 3 genome in Verticillium dahliae. (A) Investigation of the relationship of orthologues among sequenced genomes of three races of V. dahliae. Orthologues were clustered by OrthoMCL with both coverage and identities of >70%. (B) Orthologue divergence within the sequenced genome of race 3 in V. dahliae. Common orthologues were investigated using the initial pair of genomes with the highest common orthologues after introducing the sequenced genomes one by one. The number of common orthologues was determined following the introduction of the sequences from the race 3 strains of HoMCLT and GF1300. (C) Evaluation of the gene length variation of the core genes and accessory genes from HoMCLT and GF1300 genomes. Statistical significance was calculated by the gene length of indicated groups in reciprocal using an unpaired Student’s *t* tests. Total numbers of specific genes in the HoMCLT or TO22 genome and core genes were 2,401 and 7,068, respectively, as displayed in panel A.

To further understand the differentiation of race 3, the orthologue clustering genes (gene pair: coverage of >70%; identities of >70%) from races 1 and 2 were further investigated. The race 3 subclade, including strains Gf-Cb5 and GF1192, along with races 1 and 2 strains shared 1,929 specific orthologues, but a discrete subclade of race 3 strains (HoMCLT and GF1300) contained 2,401 exclusive orthologues (Fig. 2A and 3A; Table S16). Orthologue clustering of the two subclade race 3 strains with strains from races 1 and 2 supports the hypothesis that the four sequenced race 3 strains diverged such that two strains were closer to race 1 and race 2, while the other two were independent (Fig. S4). The genes in race 2 strains clustered into highest order orthologues, and the total number of orthologues was slightly reduced in the two race 1 strains and were introduced along with the two related race 3 strains, but the number was significantly reduced when the other two race 3 strains were introduced (HoMCLT and GF1300) (Fig. 3B). Interestingly, investigation of the mean gene length showed that the divergence of race 3 may be the result of accessory genes, for the mean gene lengths of core genes are similar among the JR2, HoMCLT, and GF1300 genomes, but the mean gene length from the specific orthologues of HoMCLT and GF1300 genomes are significantly shorter (1,255 bp and 1,254 bp, respectively) than the core genes (1,640 bp and 1,641 bp, respectively), with *P* values of <1.70E−54 and <1.16E−54 (Fig. 3C), respectively. This suggested that divergence of the race 3 genome was facilitated by a rapidly evolving accessory genome, consistent with the two-speed genome hypothesis (31, 32). Finally, GO annotation of the specific orthologues among the two race 3 subgroups showed that the various regulatory functions (e.g., GO:0140110 transcription regulator activity) of closely related race 1 and race 2 strains (Gf-Cb5 and GF1192) were significantly enhanced relative to the two race 3 strains (HoMCLT and GF1300) (Fig. S5 and S6). Therefore, the sequenced race 3 strains diverged into two pathotypes that display genomic and functional divergence.

### Chromosome rearrangements occurred among the three races in Verticillium dahliae.

**FIG 4:**
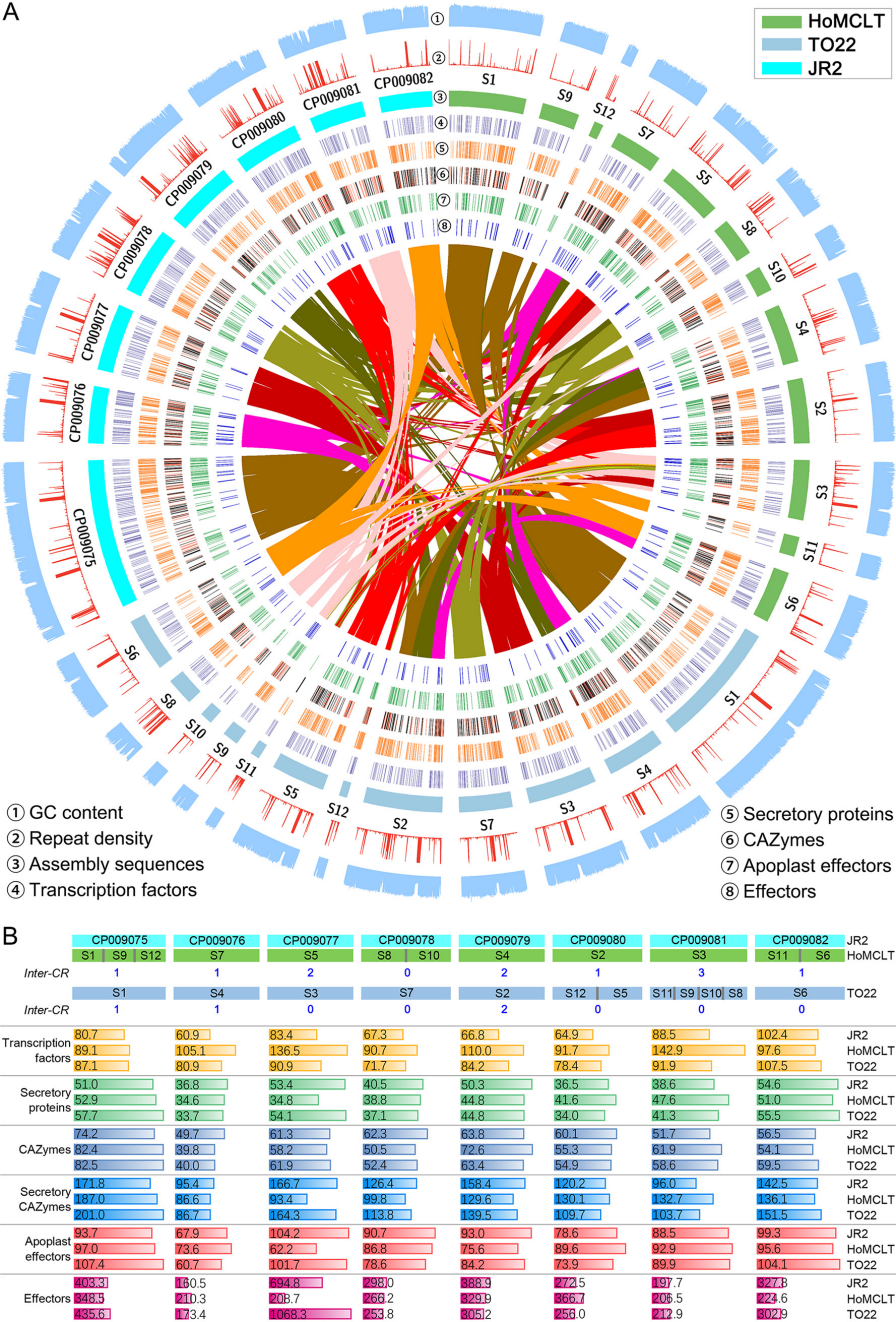
Genome structural variations among three races of Verticillium dahliae. (A) Circos diagram of the genome features among three races in V. dahliae. The subchromosomal assembly of race 3 (HoMCLT) and race 2 (TO22) was selected to construct the genome synteny with the race 1 reference genome of JR2, with 5-kb nonoverlapping windows. The order of scaffolds in the HoMCLT and TO22 genomes was determined according to the reference chromosomes in JR2. (B) Investigation of the relationship between chromosome rearrangement and the density of pathogenicity-related genes in the chromosome. Values in the colored columns represent the average sequence length (kb) in which the pathogenicity-related gene is in each chromosome.

Centromeres can contribute to chromosomal evolution through chromosomal rearrangements tied to repeats in the centromere (33). Synteny analysis of the centromeres showed that all the flanking sequence of the eight centromeres from the JR2 genome were well matched to the assembled genomes of HoMCLT or TO22, but the centromeric regions displayed significant divergence, indicating that chromosome rearrangements probably occurred during genome evolution in the TO22 or HoMCLT genome (Table S17). A previous study revealed that the evolution of the asexual pathogen V. dahliae was mediated by chromosomal rearrangements detected in the genomes of race 1 (JR2) and race 2 (VdLs.17) strains (24), and thus the potential association of chromosome rearrangements with race divergence required verification. To test this hypothesis, synteny between the assembled genomes of the three races (race 1 strain JR2, race 2 strain TO22, and race 3 strain HoMCLT) was examined. With the coordinate chromosome structure of the JR2 genome, part of the assembled sequences from races 2 and 3 did not entirely match to a single chromosome in the JR2 genome (Fig. 4A). These structural variations are typical in that sequences match to more than one chromosome (interchromosomal rearrangement [Inter-CR]). Thus, the race 3 (HoMCLT) and race 2 (TO22) genomes have at least 11 and 4 Inter-CRs compared to the reference race 1 (JR2) genome (Fig. 4B; Fig. S7 and S8A; Table S18), respectively. Investigations of the depth of the paired-end reads on the flanking sequences (±2,000 bp) of Inter-CR sites showed that the sequence assembly in Inter-CR sites is accurate (Fig. S8B), and PCR amplification confirmed that the chromosomal rearrangements occurred in each of the HoMCLT/TO22 and JR2 genomes (Fig. S8C). Synteny analyses showed that the chromosomal rearrangements were significantly associated with the repeat (transposons) density (Fig. 4A; Fig. S7), suggesting that the chromosomal rearrangements in V. dahliae may occur at transposon-rich regions, as previously suggested (24, 27). Further, analysis of the gene distribution showed that the density of pathogenicity-related genes (one gene per kilobase) was affected by the Inter-CR (Fig. 4B), although their gene numbers were similar among the three races (Table S19). Together, these results strongly suggested that the chromosome rearrangements are widespread among the races, resulting in divergence through a gene jigsaw puzzle, which may have contributed to the different biological characteristics in V. dahliae races.

### Lineage-specific regions of races in Verticillium dahliae.

**FIG 5:**
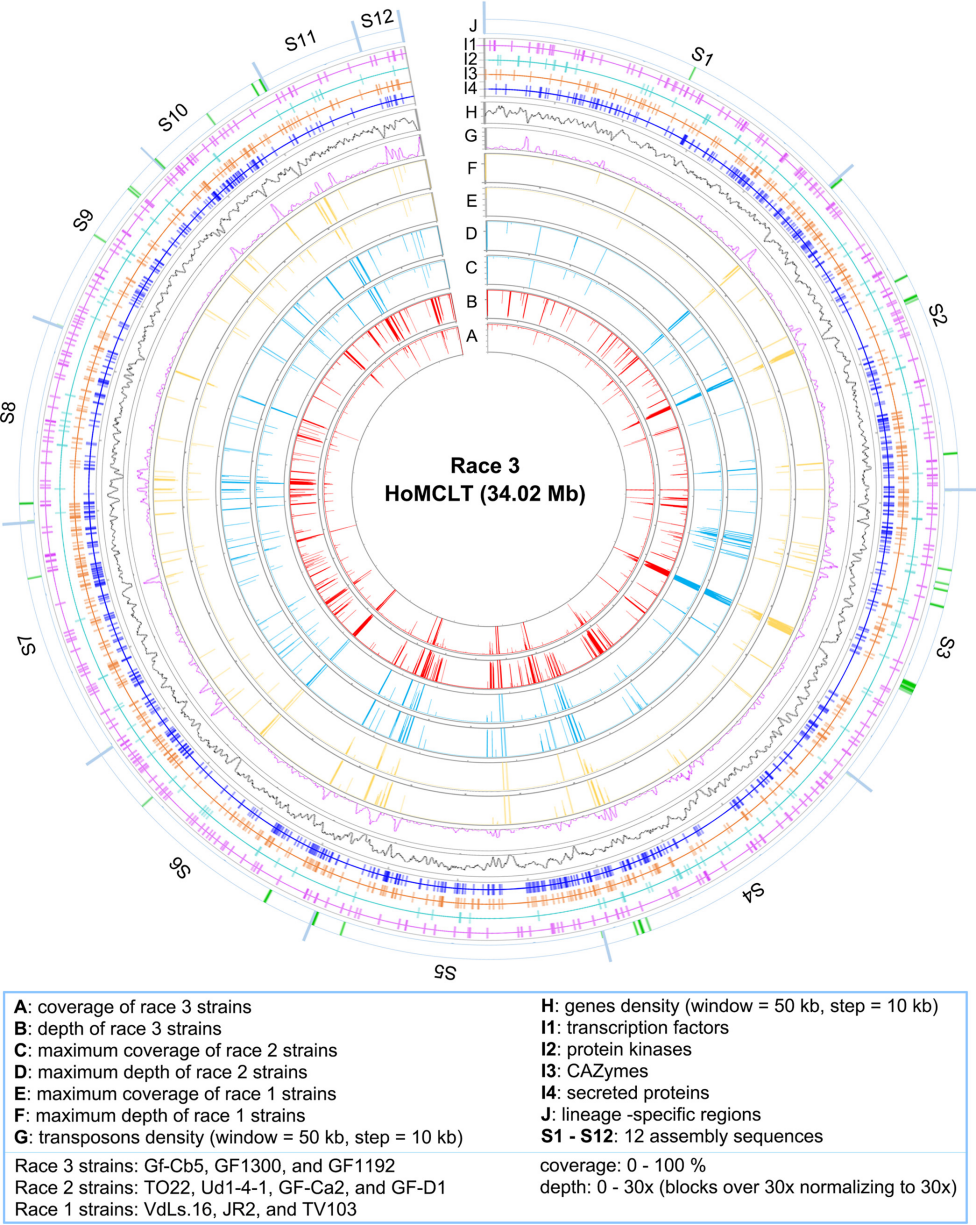
Identification of lineage-specific regions (LSRs) in the genomes of race 3 relative to race 1 and race 2 in Verticillium dahliae. The genome of race 3 strain HoMCLT was used as the reference genome, and short sequence reads of three race strains were mapped to the HoMCLT genome with a BWA program. The coverage and depth were calculated by the step window (window length: 500 bp; step: 100 bp) in each strain, and the data of read depth was normalized by 30× coverage (the depth of all windows more than 30×). The data on coverage and depth of coverage from each strain were incorporated together according to the race classification. The LSRs (green blocks in “J” circles) in the race 3 strain sequence of HoMCLT were defined by the step windows that mapped with high coverage (>50% and depth >2×) by race 3 strains but had low matching coverage with the race 2 and race 1 strains (<50%). The density of transposon sequences and genes in the HoMCLT genome was calculated by numbers that were encoded in the step window (window length: 50 kb; step: 10 kb). The short reads of the JR2 genome were download from the NCBI database (SRA: SRR515981).

Previous studies showed that the lineage-specific regions present in the genomic sequence of V. dahliae provide some genetic flexibility, and genes in the LSRs are important in conferring certain biological characteristics (24, 27). Therefore, whether the LSRs play a role in race divergence was investigated by race reference genome mapping with the sequenced reads. The assembled sequences of the HoMCLT genome were well covered by the short reads from three race 3, four race 2, and three race 1 resequenced strains (Fig. 5; Fig. S9). However, there were clearly regions that displayed lower coverage and depth when mapped with all the resequenced strains, which were significantly associated with high transposon density and low gene density (Fig. 5; Fig. S9). To investigate the regions of genetic flexibility, the reference race 3 genome (HoMCLT) sequenced reads were mapped with step windows (500-bp step windows, coverage of >50%, and depth of >2×), identifying 28 LSRs (0.7 to 126.6 kb) in total that were present in race 3 strains but absent in both race 2 and other race 3 strains (Fig. 5; Fig. S10; Table S20 and S21). Similarly, 56 LSRs (0.5 to 196.9 kb) were present in race 2 strains but absent in both race 1 and 3 strains (Fig. S10 and S11; Table S20 and S21), and 45 LSRs (0.5 to 111.6 kb, including the known *Ave1* locus [23]) were present in race 1 (R1-LS23) strains but absent in both race 2 and 3 strains (Fig. S10 and S12; Table S20 and S21). Interestingly, investigation of the read mapping efficiency showed that the short reads mapped more strongly within same race than among different races (Fig. S9, S13, and S14), which indicated that the LSRs may encompass the genetic variation associated with race divergence in V. dahliae. The distribution of pathogenicity-related genes (secreted proteins, CAZymes, TFs, and PKs) was not significantly enriched in LSRs with low gene density and high transposon density (Fig. 5; Fig. S11 and S12). Thus, the genetic flexibility conferred by LSRs therefore may also underlie the genetic basis of race divergence in V. dahliae.

### The role of lineage-specific regions among three races in host-Verticillium dahliae interaction.

**FIG 6:**
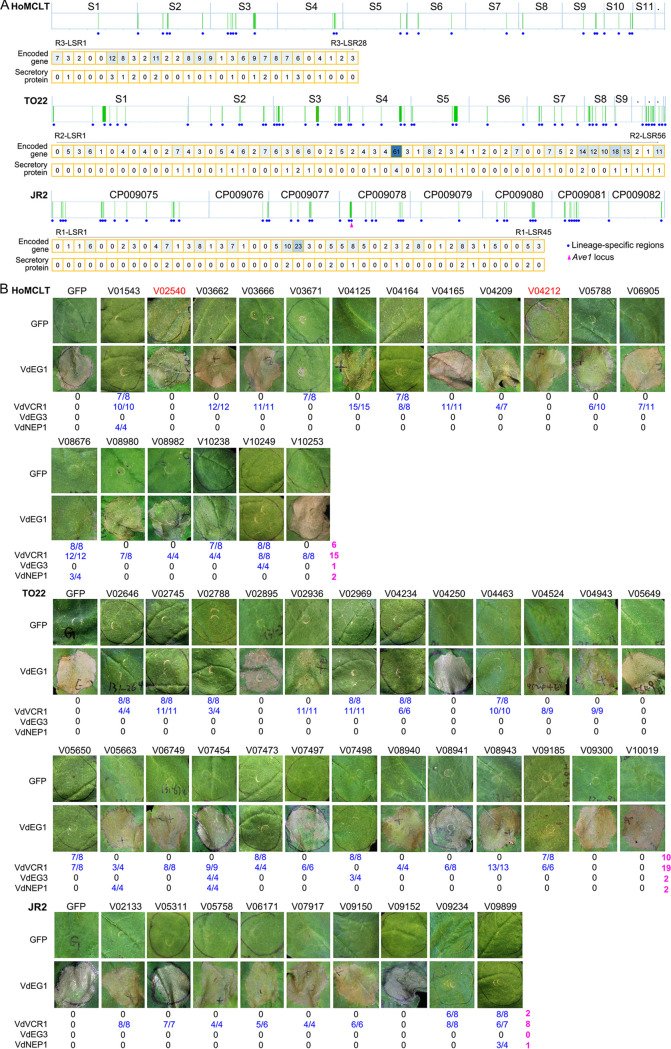
Analysis of the functions of genes encoding secreted proteins in lineage-specific regions (LSRs) among three races of Verticillium dahliae. (A) Information on genes encoding predicted secreted proteins in LSRs among three races of Verticillium dahliae. (B) The role of the genes encoding predicted secreted proteins in a host-pathogen interaction by transient expression in Nicotiana benthamiana. Cell death induction was detected for candidate genes in N. benthamiana leaves from 4-week-old plants 6 days after infiltration with Agrobacterium tumefaciens carrying the indicated genes. For immunity-suppressing activity, the candidate genes were coinfiltrated with glycosyl hydrolase family 12 (VdEG1 and VdEG3), a pathogen-associated molecular pattern (PAMP) that can induce cell death in N. benthamiana (84), cytotoxicity effector VdNEP1 (85), and the plant nucleus-dependent immunity-inducing effector VdVCR1 (unpublished data) in N. benthamiana. Infiltration of the green fluorescent protein (GFP) gene was used as a negative control. The gene number in red color represents the gene that has the ability to induce moderate cell death. The ratio number on the leaves and at the bottom of leaves represents the number suppressing cell death in the total number of leaves tested. The number in purple represents the total number of secreted proteins in lineage-specific regions (LSRs) with the ability to suppress immunity triggered by the indicated effectors.

To further examine the role of LSRs in race divergence, the functions of the genes within LSRs (including 10 kb of two flanking sequences) were investigated. In total, there were 135, 293, and 156 genes within 28 LSRs in the genome of the race 3 strain (HoMCLT), 56 LSRs from the race 2 strain (TO22), and 45 LSRs from the race 1 strain (JR2), respectively (Fig. 6A; Table S20 and S21). Functional predictive analysis of the conserved protein domains encoded by LSRs showed that only a few types were unique and displayed expansion in race 3 compared to races 1 and 2 (e.g., AMP-dependent synthetase/ligase), but several domains were unique or displayed significant expansion in races 2 and 3 (Table S22), indicating a differential enrichment of the types of TFs between the LSRs in different races (Table S23). In addition, most of the genes in LSRs of a certain race were not unique to the races, that is, orthologue clustering showed that most of the genes in LSRs clustered among races, except for 9 (6.7%), 1 (0.3%), and 20 (12.9%) genes from LSRs being unique in races 3, 2, and 1, respectively (Table S24 to S26). This indicated that some genes in the genetically flexible LSRs probably arise from a common source in V. dahliae. Finally, many genes within LSRs encode proteins of unknown function among the races (around 30% of genes without conserved domain annotation; 50, 118, and 43 genes among races 1, 2, and 3, respectively). This significantly restricts the understanding of their potential roles in race divergence in V. dahliae (Table S24 and S25).

Secreted proteins play important roles in host-pathogen interactions, including those proteins encoded by the LSRs in V. dahliae (24). Of the genes within LSRs among genomes of the different races, 20 (14.8%, 12 members with signal peptide), 27 (9.2%, 18 members with signal peptide) and 13 (8.4%, 10 members with signal peptide) genes from race 3, race 2, and race 1, respectively, were predicted as secreted proteins (extracellular location without transmembrane) (Fig. 6A; Table S23). To further understand the role of the secreted proteins of the LSRs in host-pathogen interactions, the activity of potential effectors (length less than 500 amino acids; 18, 25, and 9 candidates from races 3, 2, and 1, respectively) to induce or suppress immunity was assayed by transient expression in Nicotiana benthamiana. Interestingly, the potential secreted protein encoded in LSRs, especially from race 2 and race 3 strains, revealed the striking function of differentially suppressing immunity induced by several known effectors from V. dahliae (Fig. 6B). In addition, two potential effectors from race 3 (HoMCLT_V02540 and HoMCLT_V04212) induced moderate cell death in tobacco (Fig. 6B). Together, these results suggest that the secreted proteins in LSRs play critical roles during host-V. dahliae race-specific interactions to suppress host plant immunity and facilitate infection.

### Race 2- and 3-specific effectors contribute to pathogenesis on tomato.

**FIG 7:**
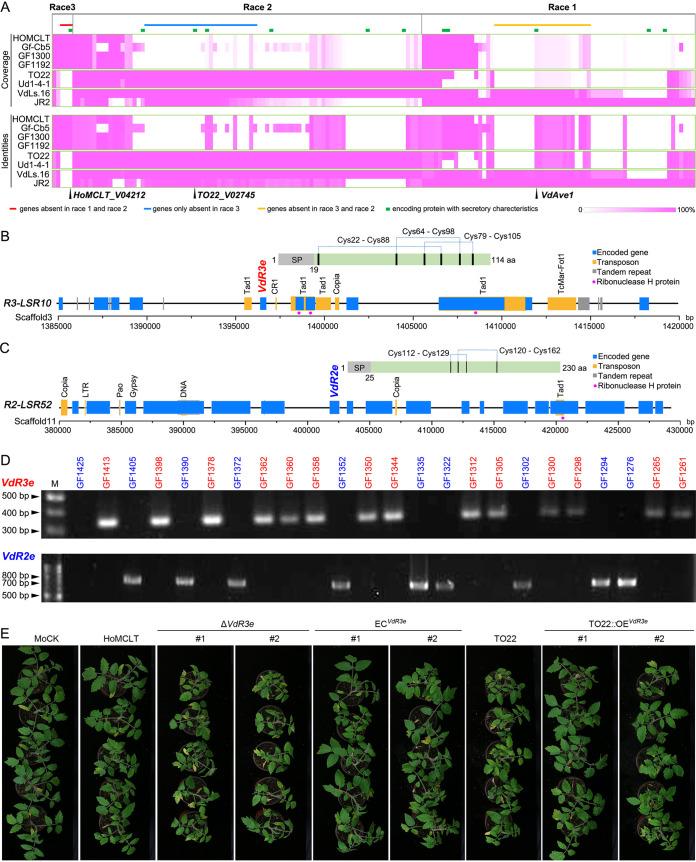
The role of specific effectors in races 2 and 3 in the pathogenesis of Verticillium dahliae. (A) Identification of the specific effectors from the genes in lineage-specific regions (LSRs). The genes from LSRs in one race were collected and compared to those from the genomes of two other races by BLASTN (E value of <1E−10). Genes with the identities of >90% or sequence coverage of >90% were excluded. The identities and coverage of each of the genes were investigated among three races, and the genes only present in one race but absent in the other two races were collected (for race 2, only the genes absent in race 3 were collected because the avirulence factor of race 2 generally accompanies the Ave1 avirulence factor of race 1). Green boxes represent the gene encoding a predicted secreted protein. (B, C) Diagram of the candidate race 2- and race 3-specific loci and the deduced protein structure of candidate race-specific proteins; Cys, cysteine residue. Race 3-specific factor VdR3e locates in the LSR of R3-LSR10 (B). Race 2-specific factor VdR2e locates in the LSR of R2-LSR52 (C). (D) PCR assay for the race 2 VdR2e or race 3 VdR3e in the Verticillium dahliae population. The population was collected from tomato in Japan, including strains of races 2 and 3 that have been race verified by pathogenicity tests on tomato. The strain ID with blue and red color represents the race 2 and race 3 strains, respectively. (E) Pathogenicity assay of candidate race 3 avirulence factor deletion mutant (VdR3e) of V. dahliae. Two-week-old tomato (resistance tomato germplasm IVF6384) seedlings were root-dip inoculated with the two independent *VdR3e* deletion strains, and two complementary transformants of reintroducing *VdR3e* with the HoMCLT background, two transformants of overexpression *VdR3e* under the race 2 background TO22 strains, race 3 strain HoMCLT, and race 2 TO22 were used as the positive controls. Roots inoculated with sterile water were used as negative controls. The tomato plants were photographed at 21 days after inoculation.

Race 1 in the V. dahliae*-*tomato interaction is determined by the expression of avirulence factor *Ave1* that is recognized by the tomato plant, which in turn mounts an effective defense response, rendering race 1 of V. dahliae nonpathogenic (23). *Ave1* locates in the R1-LSR23 in the JR2 genome (Fig. 6A). Comparison of the LSR gene sequences showed that only five genes of the HoMCLT genome are specific to the genomes of the TO22 and JR2 strains, similar to the 87 genes from the LSRs of the TO22 genome and 68 genes from the LSRs of the JR2 genome being specific to the genomes of these two strains (Fig. 7A; Table S27). Of the five genes in the HoMCLT genome, only three were present in race 3 when the genomes of three races were compared, and one (*HoMCLT_V04212*) was predicted as a secreted protein with characteristics of an effector (Fig. 7B; Table S27) and was endowed with the name V. dahliae race 3 specific-effector (VdR3e). PCR assays showed that *VdR3e* was only present in race 3 strains but was absent in race 1 and 2 strains (Fig. 7D; Table S28), further suggesting that VdR3e is a secreted protein specific to the race 3 population. Unexpectedly, no specific genes were identified in race 2 when the additional genomes of three races were compared (Fig. 7A; Table S27). A previous study showed that the race 2 avirulence factor frequently accompanies the race 1 avirulence factor VdAve1 (34). Therefore, the presence of 87 genes (from the LSRs in TO22) in the genomes of both race 2 and race 1 but absent in the genomes of race 3 was investigated, leading to the identification of two genes (*TO22_V04943* and *TO22_V02745*) that encode products characteristic of secreted proteins (Fig. 7C; Table S27). PCR assays showed that only *TO22_V02745* (named V. dahliae race 2 specific-effector [VdR2e]) but not *TO22_V04943* is absent in the race 3 population (Fig. 7D), which suggested that *VdR2e* is a candidate effector of the race 2 population.

*VdR3e* is located in R3-LSR10 of the HoMCLT genome (∼35-kb specific region) in a genetically flexible, transposon-rich region (*Tad1*, *Copia*, etc.) (Fig. 7B). Moreover, *VdR3e* is a singleton exon that was predicated to encode a typical effector (114 amino acids) with six cysteine residues that may form three disulfide bonds (Fig. 7B) and has the ability to induce cell death in tobacco (Fig. 6B), indicating that *VdR3e* may play a critical role in the plant-V. dahliae interaction. To clarify the role of this putative effector of the race 3 strain in pathogenesis, we screened the tomato germplasms resistant to race 3 but susceptible to race 2 (Fig. S15; Table S29) and randomly selected the tomato germplasm IVF6384 for additional research. Interestingly, deletion of the *VdR3e* enhanced the aggressiveness of V. dahliae because the deletion mutant caused more severe disease symptoms and accumulated more fungal biomass than the wild-type race 3 strain HoMCLT in tomato; conversely, the race 2-susceptible tomato germplasm was resistant when *VdR3e* was introduced into race 2 strain TO22 (Fig. 7E; Fig. S16A). Deletion of *VdR3e* did not affect the virulence of race 3 strains on susceptible tomato (cv. Momotaro and cv. MoneyMaker) (Fig. S17). Similarity, *VdR2e* is located in R2-LSR52 of the TO22 genome (∼49-kb specific region), which also is surrounded by several transposons (Fig. 7C). Moreover, the encoded protein (230 amino acids) of *VdR2e* contains four cysteine residues that may form two disulfide bonds (Fig. 7C), and suppresses the immunity caused by the effectors VdEG1 or VdHnuc on tobacco (Fig. 6B). Unexpectedly, the resistance of tomato (cv. Aibou) against race 2 strains remained following the deletion of *VdR2e* in the race 2 strain TO22 (Fig. S18). However, the pathogenicity assay on susceptible tomato showed that deletion of the *VdR2e* in race 2 strain TO22 compromised virulence on cv. MoneyMaker but not cv. Momotaro (Fig. S18), suggesting that VdR2e acts as the immunity suppressor that differentially contributes to virulence on tomato. Taken together, these results indicate that V. dahliae recruits specific effectors VdR3e and VdR2e to contribute to virulence on tomato that delineate different races.

## DISCUSSION

The study of the evolution of pathogen races has been an eternal theme in plant pathology for effective disease management. Ever since the first races of V. dahliae were described in tomato (10–12), and subsequently in lettuce (17), race structure has not changed, and cultivars with resistance to race 2 have not been developed and released. In 2017, however, the original race 2 was split into races 2 and 3 based on the response of the differential tomato rootstock cultivar Aibou that exhibited resistance to race 2 (18). In this study, by sequencing the genomes of different races, we systematically investigated the lineage-specific regions (LSRs) in races and their functional role of encoding genes in race divergence. A few secreted proteins in LSRs differentially suppress the immunity triggered by known effectors, contributing to the differential virulence of the races. Of these, the race 2- and 3-specific effectors (*VdR2e* and *VdR3e*, respectively) contribute to virulence in races 2 and 3 on tomato, and *VdR3e* is an avirulence factor of race 3.

Currently, more than 20 V. dahliae genomes have been released, but the genetic basis of race divergence has not been elucidated. Here, we sequenced the genomes of two race 2 and four race 3 strains and compared these sequences with those of two previously released race 1 genomes (24, 30). Genome characteristics of V. dahliae among the three races were similar but showed significant divergence in their LSRs, which serve as a repository of biological characteristics in V. dahliae. Potential effectors located in the LSRs in all three races suppressed immunity in host-pathogen interactions (Fig. 6). We identified potential effectors, each specific to race 2 (confirming the discoveries of Chavarro-Carrero et al.) (26) and race 3, that can be used to identify the two races in the pathogen populations from hosts that lack homologs of the resistance gene *V2*.

The description of the first V. dahliae genome (VdLs.17) explored the genetic basis of why this pathogen colonizes and proliferates in the unique vascular niche, its enhanced capacity to degrade plant materials, and the importance of LSRs (27). Subsequently, using the genome of a race 1 strain from tomato (JR2) and a clever combination of comparative genomics and functional assays, the first avirulence gene (*Ave1*) was identified (23). Furthermore, the evolutionary mechanisms used by predominantly asexual pathogens through extensive chromosomal reshuffling was delineated (24). Using a comparative genomics approach and functional assays, Chavarro-Carrero et al. identified a 277-kb race 2-specific sequence that encoded predicted secreted proteins (26). The two genes in this region were both expressed by V. dahliae during tomato colonization. However, functional analysis revealed that only one of the two genes served as the avirulence effector Av2 that is recognized in *V2* tomato plants. With the exception of identifying the avirulence factors associated with races 1 and 2, the genetic basis of race divergence in V. dahliae remained undetermined. Several pathogenicity-related factors encoded by genomes are generally used for describing the biological characteristics, including secreted proteins, small cysteine-rich proteins (SCRPs), CAZymes, pathogen-host interaction (PHI) homologs, and PKs (28). In V. dahliae, the expansion of CAZymes (enhanced pectinolytic machinery) is a unique characteristic that may enable it to proliferate in the plant vascular system (27), and these pathogenicity-related factors may be involved in race divergence as well. Unexpectedly, the similarity of these pathogenicity-related factors, even at the subfamily level, are quite high among the genomes of the three races (Fig. 1; Table S6 to S10). Therefore, our results suggested that the content and composition of the basic machinery for colonization and proliferation are highly conserved in all three races of V. dahliae.

Comparative genomics also revealed that the flexible genomic islands in V. dahliae that contain duplicated genes play critical roles in signaling/transcriptional regulation (27), suggesting that genes involved in signaling/transcriptional regulation may be the drivers of pathogenicity/virulence evolution in V. dahliae. The TFs, as key factors in transcriptional regulation, have long been recognized as the critical proteins for fungal pathogenicity, as many are known to play important roles in the transcriptional regulation of pathways implicated in virulence (35). Several previous studies have proven that TFs play critical roles during V. dahliae infection of the host plant (36). The content and composition of TFs display significant divergence among the three races in V. dahliae, and the total number of TFs is lower in the race 3 genome than in the genomes of either race 2 or race 1. The two race 3 genomes (HoMCLT and GF1300) encode around 100 fewer TFs than the other genomes (Fig. 1; Table S11). Orthologue analysis among the genes of the three races further suggested that genes with binding function show enhanced divergence in the race 1 genomes (Fig. 2C; Table S13). Therefore, the divergence of transcriptional regulation, especially in the TFs, is potentially a key driver of race evolution in V. dahliae.

Because V. dahliae is a strictly asexual pathogen with little or no evidence of recombination (24), it is difficult for the pathogen to accumulate beneficial mutations by clonal reproduction alone, especially in the core genomes of the V. dahliae population. Therefore, the pathogen probably uses the LSRs to act as a reservoir to deposit sequences with novel functions for the evolution of certain biological characteristics (37). As described above, V. dahliae possesses several LSRs that are important in signaling/transcriptional regulation and iron/lipid metabolism, which likely facilitate pathogen colonization and proliferation (27). Similarly, the avirulence genes *Ave1* and *Av2* were also located in the LSRs that define the properties of race 1 and race 2 (23, 26), respectively. In contrast, the genomes of defoliating and nondefoliating strains contain genes clustered within a single LSR that specify defoliation functions on the host plants caused by the defoliating strain (28, 29). Several race independent LSRs among races also occur and possess hundreds of genes (Table S20). The predicted functions of these genes in LSRs are also a characteristic of race divergence (Table S21 and S22) corresponding with the orthologue analysis (Fig. 2C). For instance, the LSRs of race 3 encode more genes involved in energy and metabolite biosynthesis, while genes involved in transcription are enriched in the LSRs of race 2 and race 1 (Table S21 and S22). TFs regulate several cell processes, including adaptation to environmental stress and interactions with the host immune system (35). Therefore, except for the evolution of avirulence genes, the genetic basis of race divergence is likely mediated by the genes involved in transcriptional regulation in V. dahliae.

Secreted proteins play crucial roles in the interactions between pathogens and plants to promote successful colonization, proliferation, and infection (38). The secreted proteins located in the LSRs are important for pathogenesis and manipulation of host immunity (39, 40). In V. dahliae, the secreted proteins encoded in the LSRs determine race divergence, including *Ave1* in race 1 and *Av2* in race 2 (23, 26). Many plant-induced effectors in V. dahliae are present in the LSRs and contribute to pathogenesis (24, 41, 42). Our results also showed that many potential secreted proteins are in the LSRs among the three races (Fig. 6A). As expected, most of them are involved in host-pathogen interactions; two from race 3 have the ability to induce immunity, and several others suppress immunity to facilitate pathogenesis of V. dahliae (Fig. 6B).

In the tomato-V. dahliae pathosystem, the pathogen can be grouped into two races based on response to a single dominant resistance locus called *Ve*. Isolates that are avirulent on tomato carrying *Ve* are considered race 1 (9), and those that escape recognition and cause the disease are grouped as race 2 (10, 11, 12). In our study, comparison of orthologues showed that the genomes also displayed significant divergence within the four race 3 strains (Fig. 2 and 3). The gene content and structure of the four race 3 strains diverged into two types (Fig. S2). These results suggest that the current race 3 population likely encompasses two different pathotypes, and these can only be phenotyped as different races when tomato germplasm resistant to one of these is available. Indeed, the race scenario in V. dahliae is far more complex than is currently known. For instance, in the cotton-V. dahliae pathosystem, a few studies had previously shown that V. dahliae can be separated into five races based on virulence tests on several cultivars (43–45). Race determination by race-specific markers also confirmed that the strains from cotton in China cannot be assigned to the current three races (Table S28). Therefore, the analyses of race divergence among populations remains a challenging but fertile area of research.

Previous studies showed that extensive chromosomal rearrangements may drive the evolution of V. dahliae because it is an asexual pathogen. Among the three released genomes (JR2, the race 1 strain, and the two race 2 strains [classification in traditional method] VdLs.17 and Vd991), chromosomal rearrangements are frequently observed (24, 28). In this study, extensive chromosomal rearrangements were documented among the selected genomes from the three races (Fig. 4). In addition, the centromeric regions displayed significant divergence in the genomes, and the two flanking sequences of centromeres from the JR2 genome matched to the different assembled sequences in the HoMCLT genome (Table S17). A previous study showed that centromeres contributed to chromosomal evolution because the centromeres could be linked chromosomal rearrangements in V. dahliae (33). This phenomenon indicates that the chromosomal rearrangements may be associated with race divergence in V. dahliae. However, the chromosomal structure of two race 1 genomes (JR2 and VdLs.16) also exhibited extensive chromosomal rearrangements (Fig. S7D). Therefore, chromosomal rearrangement may be a mechanism that underlies race divergence via the generation of novel genes, such as *Ave1* (24), but does not by itself define race structure in V. dahliae.

Interestingly the genome of race 3 diverged into two groups with strikingly different gene lengths (Table 1; Fig. S2), and this divergence (shorter gene features) was mainly caused by the accessory genes from the HoMCLT and GF1300 genomes (Fig. 3C). The two-speed genome is a fundamental characteristic of rapidly evolving pathogens whereby housekeeping genes reside in a conserved core genome, and pathogenicity-related genes reside in dynamic and repeat-rich compartments (31, 32). Although LSRs display markedly higher sequence conservation in coding as well as noncoding regions than the core genome among the species of *Verticillium* (42), the potential effectors in the highly variable regions are enriched in genes that determine races and operate under the two-speed genome evolution in V. dahliae (46, 47). In this study, we found that the gene features of race 3 strains displayed significant divergence between the accessory and core genomes (Fig. 3C), suggesting that the divergence within race 3 strains could be driven by two-speed genome evolution. More genome investigations of the different gene features between the accessory and core genomes may further demonstrate this unique feature of genome evolution in V. dahliae.

## MATERIALS AND METHODS

### Isolate collection, cultures, and DNA preparation.

All isolates of V. dahliae used in this study were collected from infected tomato plants in Japan and were typed to races based on the response of differential tomato cultivars (Table S1). The isolates were single spored from cultures on potato dextrose agar (PDA). Cultures from each isolate were grown in 250 mL of complete medium (CM; yeast extract, 6 g/L; casein acid hydrolysate, 6 g/L; sucrose, 10 g/L) at 25°C, and fresh mycelia were collected after 5 days of growth in a shaker set at 110 rpm. High-quality genomic DNA was extracted using the Quick-DNA fungal/bacterial midiprep kit (Zymo Research, Orange, CA, USA) according to the manufacturer’s protocol.

### Library construction and sequencing.

For each isolate of V. dahliae sequenced, two libraries with insert sizes of 20 kb and 270 bp were constructed using SMRTbell template prep kits (Pacific Biosciences, Menlo Park, CA, USA) and Illumina TruSeq Nano DNA library prep kits (Illumina, San Diego, CA, USA), respectively. In detail, genomic DNA (10 μg) was mechanically sheared using a Covaris g-Tube (KBiosciences, 520079) with a goal of DNA fragments of approximately 20 kb. The fragment size distribution was assessed using a Bioanalyzer 2100 12K DNA chip assay (Agilent, 5067-1508). The 20-kb SMRT Bell library was prepared using a DNA template prep kit 1.0 (PacBio, 100-259-100). A blunt-end ligation reaction followed by exonuclease treatment was performed to generate the SMRT Bell template, and the enriched large fragments (>10 kb) were selected using the Blue Pippin device (Sage Science, Inc., Beverly, MA, USA). The size-selected library was inspected for quality and quantified on an Agilent Bioanalyzer 12 kb DNA chip (Agilent Technologies, Santa Clara, CA, USA) and a Qubit fluorimeter (Invitrogen, Carlsbad, CA, USA). A ready-to-sequence SMRT Bell-polymerase complex was created using a Binding kit 2.0 (PacBio, 100-862-200), according to the manufacturer’s instructions. The Sequel instrument was programmed to load and sequence the sample on PacBio SMRT cells v3.0 (PacBio, 100-171-800), acquiring one movie of 360 min per SMRT cell on the PacBio RS II platform. The MagBead loading (PacBio, 100-125-900) method was used to improve the enrichment of the larger fragments. The short insert size of a 270-bp library was sequenced on an Illumina HiSeq 2000 instrument at Beijing Genomics Institute (Shenzhen, Guangdong).

### Filtering of the sequence data.

For the PacBio data, subreads were filtered by the following parameters: filtered subreads with adapters, removed the quality of polymerase reads less than 0.8, and filtered the length of subreads less than 1,000 bp. For the Illumina data, the clean reads were filtered by the following parameters: filtered reads with adapters, trimmed reads with two low-quality bases at the 5′ end and three low-quality bases at the 3′ end, removed reads with *N* bases more than 10%, filtered duplicated reads due to PCR amplification, and discarded reads with low-quality bases (≤5) greater than 50%. After removing adaptor sequences, 34 million short clean reads (>4.0 Gb) and 3.4 million subreads (>5.0 Gb, with a subread mean length of >9.5 kb) for each isolate were obtained by Illumina and PacBio sequencing (Table S2), respectively.

### Genome assemblies.

The genome sequences were assembled *de novo* using the PacBio subreads with the SMARTdenovo program and the designated parameters (-c 1 -t 8 -k 16) and extended by the SSPACE program using subreads (parameters: -o 50 -l 3) and Illumina clean reads (parameters: -o 20 -m 32 -k 5 -n 15) (48). The PBjelly2 program (49) was used for minding the gap of assembled sequences with the default parameters. Error corrections were performed using subread by the variantCaller program from the SMRTlink v4 package (Pacific Biosystems). Subsequently, the GATK program (50) was used for further error corrections with the designated parameters (-cluster 2 -window 5 -stand_call_conf 50 -stand_emit_conf 10.0 -dcov 200 MQ0 ≥ 4). The assembled sequences were corrected by the SOAPsnp (parameters: -u -t -z @ -Q i -q) and SOAPindel programs (parameters: -c 3 -h 1 -u 2 -m 2) (51) using Illumina clean reads.

### Gene prediction and annotation.

Protein-coding genes in all the sequencing genomes were predicted using a combination of *de novo*-based and homology-based approaches, as described previously (28). For the *de novo* prediction, the gene prediction of repeat-masked genomes was implemented by GeneMark-ES (52) together with V. dahliae (VdLs.17 and VdJR2)-trained Augustus (version 2.6) (53) and the SNAP program (54). For the homology-based prediction, the protein-coding genes of VdLs.17, VaMs.102 (*V. alfalfae*, previously named “*V. albo-atrum*”) (27), JR2 (24), and representatives from three phenotypically diverse species/species complexes of Fusarium (F. graminearum, F. verticillioides, and F. oxysporum) (55), Nectria haematococca (=F. solani species complex mating population VI) (56), and Magnaporthe oryzae (57) were collected and mapped onto the Vd991 genome using TBLASTN. Homologous sequences aligned to the matching proteins were defined as gene models of Vd991 using the GeneWise program (58). Finally, all gene evidence was combined using GLEAN (59). The general annotation of predicted proteins was performed with the following programs. Putative functional annotations were interrogated to known databases using BLASTP to identify the best homologues, including the databases of nr, eggNOG (60), and InterProScan (incorporated InterPro, GO, and KEGG pathway annotation) (61).

### Characterization of transposons.

Transposable elements were identified by RepeatMasker (open 3.2.8, detailed parameters: -no_is, -norna, -engine, -s, -parallel = 1, used Repbase version 15.08) and RepeatProteinMask (-noLowSimple, -pvalue = 1e-4) (http://www.repeatmasker.org). The output files were summarized using a custom Perl script.

### Orthologue analysis.

The orthologue groups among all sequencing isolates were clustered by two strategies of OrthoMCL (51) and reciprocal best hits (RBH) using the BLASTP program (62). For OrthoMCL analysis, a set of high-quality gene models was obtained by rejecting low-quality sequences (shorter than 10 amino acids, >20% stop codons, and >20% nonstandard amino acids). Pairwise sequence similarities between all input protein sequences were calculated by all-by-all BLASTP (parameters: E value of <1E−5 and >70% match length); subsequently, a Markov clustering algorithm was applied with an inflation value (−I) of 1.5 (default value in OrthoMCL) for defining orthologue cluster structure. For clustering by RBH strategies, reciprocal BLAST analysis of the genes among all sequenced genomes was performed using the BLASTP program (E value of <1E−5) to find all pairwise matches. The Solar software (version 0.9.6, http://sourceforge.net/p/treesoft/code/HEAD/tree/branches/dev/) was used to remove redundant members with progressive parameters (both match rate and identities are less than 70%). The pairwise matches from the BLAST results were clustered using the clustering application Hcluster_sg (63) for the orthologues among all the sequenced genomes. The encoded proteins of *Verticillium alfalfae* were used as an outgroup. The orthologous relationships among the sequenced genomes or three races were drawn by VennPainter (64). Significant GO catalogs of the response orthologues were selected by the Pearson chi-square test (*P < *0.05) using the WEGO tool (65).

### Functional annotation.

**(i) Secretomes.** Secretory proteins of all sequencing genomes were identified using four programs, as described previously (27). The WoLF PSORT software (fungi model) was used for the subcellular localization of all encoding proteins (66); signal peptides and signal peptide cleavage sites of putative extracellular proteins were predicted using the SignalP software (version 4.1; d-Score cutoff set to 0.500) (67); all putative extracellular proteins with signal peptides were then analyzed for the presence of transmembrane domains using TMHMM 2.0 (68) and Phobius (69) software to identify protein sequences containing a signal peptide but lacking transmembrane domains; the proteins characterized as extracellular and containing a signal peptide but lacking transmembrane domains were identified as secreted proteins.

**(ii) Small cysteine-rich protein-type effector.** Based on the secretome set of predicted proteins, the length of the peptide and the number of cysteine residues were calculated by using a custom Perl script, and those proteins with <400 amino acids and ≥4 cysteine residues were designated a SCRP-type effector.

**(iii) Carbohydrate-active enzymes.** The annotation of putative CAZymes was performed using the hidden Markov model (HMM)-based routine of the carbohydrate-active enzymes database (70). Significant hits compared to the CAZymes database were analyzed in the set of putative CAZymes using BLAST (E value of <1E−5 and identities of >30%) and were used to increase the accuracy of the CAZyme annotation. CAZymes involved in plant cell wall degradation were collected according to the classification methods in previous publications (71, 72).

**(iv) Pathogen-host interaction factors.** The homologs of known pathogen-host interactions factors were predicted using PHI-base (version 4.7, http://www.phi-base.org) (73).

**(v) Protein kinases.** PKs were predicted by running HMM searches locally with Kinomer (version 1.0) (74).

**(vi) Transcription factors.** TFs were designated by the conserved domains that were predicted through use of the InterPro database.

**(vii) Effectors.** All predicted proteins were screened by ApoplastP (75) and EffectorP software (76). The candidate apoplastic effectors and effectors were further filtered for secretion as described above.

### Phylogenetic and evolutionary analysis.

The MAFFT program (LINSi; v7.04b) (77) was applied for sequence alignment for each individual orthologue and subsequently concatenated. The phylogeny tree was constructed using RAxML (v8.2.4) by the maximum likelihood method with the GAMMA model of rate heterogeneity and the Whelan and Goldman (WAG) model of amino acid substitutions (78). There were 1,000 rapid bootstrap approximations.

### Identification of chromosomal rearrangement.

The genomes of HoMCLT, TO22, and JR2 were selected for genome structure comparison among three races. The whole-genome alignments were generated by the NUCmer program from the MUMmer 3.0 package (79) using the high-coverage parameter setting (-b 5000 -c 500 -l 200 -g 10000). According to the whole-genome alignments generated by the NUCmer program, the chromosomal rearrangements between genomes were identified by the assembled sequences of one genome that matched different physical locations on another genome using Perl scripts. Colinear blocks, repetitiveness, percent GC, and distribution of pathogenicity-related genes (including secretory proteins, SCRPs, CAZymes, and effectors) in the sequenced genomes were visualized using Circos (version 0.55) (80).

Chromosomal rearrangement sites were validated by the depth of the paired-end reads mapping. In detail, the paired-end reads were mapped to the flanking sequences (± 2,000 bp) by the BWA program (-o 1 -e 63 -i 90 -L -k 2 -l 31 -t 4 -q 10) (81), and the depth of each base in the chromosomal rearrangement sites was calculated by mapping group reads. For PCR validation, the primer pairs were designed across the chromosomal rearrangement sites in the HoMCLT genome and their synteny blocks in the JR2 genome, and the following PCR cycling parameters were used: 95°C for 5 min, (95°C for 15 s, 60°C for 15 s, 72°C for 30 s) × 25, 72°C for 5 min. Primers are listed in Table S30 in the supplemental material.

### Identification of the lineage-specific regions among races.

The genomes of HoMCLT, TO22, and JR2 were set as references for race 3, race 2, and race 1 to identify the lineage-specific regions (LSRs), respectively. With the reference genome, the short reads of all sequenced strains were aligned by the BWA program (-o 1 -e 63 -i 90 -L -k 2 -l 31 -t 4 -q 10) (81). The read coverage and depth of each strain matched to the step window (window length: 500 bp; step: 100 bp) in the reference genome were calculated. The data of read depth were normalized by 30× (the depth of all windows was more than 30×). The LSRs of each race were defined such that the step window was well mapped (coverage of >50% and depth of >2×) by the short read from same race strains but low match (coverage of <50%) or absent among another two races strains.

### PCR validation of the race-specific genes.

The genes of LSRs were collected from race 3 (HoMCLT) or race 2 (TO22) genomes. BLASTn (E value of <1E−10) was used for sequence-specific detection of these genes that are only present in one race but absent in the other two races. The candidate genes associated with races were detected by PCR (95°C for 5 min, [95°C for 15 s, 60°C for 15 s, 72°C for 30 s] × 25, 72°C for 5 min) using DNA from race 2 and race 3 populations from tomato collected in Japan (18). Primers are listed in Table S30.

### Transient expression in Nicotiana benthamiana.

The V. dahliae genes were amplified from the cDNA of strains HoMCLT and TO22 and cloned separately into the PVX vector pGR107 with the ClonExpress II one-step cloning kit (Vazyme, Nanjing, China) according to the manufacturer’s instructions. The recombinant plasmid was transformed into Agrobacterium tumefaciens strain GV3101. A. tumefaciens cells carrying the tested genes were grown in Luria-Bertani (LB) medium at 28°C overnight. Cells were harvested and washed in salt solution with 10 mM MgCl_2_, 10 mM morpholineethanesulfonic acid (MES), and 200 μM acetosyringone, pH 5.6 and resuspended to an optical density at 600 nm (OD_600_) of 0.8 for the assays of cell death induction. To examine the suppression of cell death induction in N. benthamiana, two A. tumefaciens cells carrying appropriate constructs were mixed at a 1:1 ratio to an OD_600_ of 0.8 for each and for coinfiltration. The transient expression assays were performed in 4-week-old N. benthamiana plant leaves using Bcl-2-associated X protein (BAX) and green fluorescent protein (GFP) as positive and negative controls, respectively. Symptom development was monitored at 3 days in a time course experiment until 6 days postinfiltration (dpi).

### Screening of resistance in tomato germplasm.

Tomato (*Solanum lycopersicum*) plants were grown in the greenhouse at 25°C during 16-h/8-h day/night periods. In the first stage inoculation test, 50 tomato germplasm lines (Table S29) were used for resistance identification to V. dahliae race 3 strain HoMCLT. The inoculation experiment was performed according to the previous method (82) with a few modifications. Five 3-week-old tomato seedlings were inoculated with V. dahliae race 3 strain HoMCLT by dipping roots into a 1 × 10^7^ conidia/mL spore suspension for 20 min. Inoculated and noninoculated tomato seedlings were planted in soil and grown in a greenhouse. Disease symptoms were observed at 3 weeks postinoculation.

Tomato germplasm types resistant to V. dahliae race 3 strain HoMCLT were used in the second stage inoculation test. Three or four seedlings of each tomato germplasm were inoculated with three V. dahliae race 3 isolates (GF1202, GF1300, and GF1358) and one race 2 isolate (TO22), respectively. The inoculation method and observations of disease symptoms were performed as described above.

In the third stage inoculation test, representative isolates of three V. dahliae races (GF1259 and GF1192 for race 3, GF1335 and GF1276 for race 2, JR2 and 307 for race 1) were used for resistance and susceptibility identification of the specific tomato germplasm against V. dahliae race 3. The inoculation method and observations of disease symptoms were performed as described above.

For biomass quantification, rhizome tissues were collected and used for total DNA isolation. Quantitative PCR (qPCR) was performed with the primer pairs SIRUB-F/R for tomato *RuBisCo* and VdITS1-F/VdSTVe1-R for the V. dahliae internal transcribed spacer (ITS) listed in the Table S30. The following qPCR conditions were used: an initial 95°C denaturation step for 5 min, followed by denaturation for 30 s at 95°C and annealing for 30 s at 60°C and extension for 30 s at 72°C for 40 cycles.

### Fungal transformations.

For gene deletion of *VdR3e* from strain HoMCLT or *VdR2e* from strain TO22, the targeted gene deletion construct was generated comprising the approximately 1-kb flanking regions of *VdR3e* or *VdR2e* in vector pGKO2-*Hyg* using primer pairs pGKO2-VdR3e-Up-F/R and pGKO2-VdR3e-Dn-F/R for strain HoMCLT and primer pairs pGKO2-VdR2e-Up-F/R and pGKO2-VdR2e-Dn-F/R for strain TO22 (Table S30). To generate complementation transformants, the sequence, including the native promoter region, gene sequence, and native terminator region of the targeted gene, was amplified using the primers EC_VdR3e-F/R or EC_VdR2e-F/R (Table S30) and was cloned into the donor vector pCOM that carries Geneticin resistance. Similarly, a fused fragment comprising the *TrpC*-promoter region, coding sequence of *VdR3e*, and *Nos* terminator was introduced into the vector pCOM (83).

All positive recombinant vectors were transferred into A. tumefaciens strain AGL-1 for fungal transformation. Gene-knockout mutants and complement transformants were generated by the A. tumefaciens-mediated transformation (ATMT) method described previously (41). The knockouts and complemented transformants were selected and isolated on PDA medium with specific antibiotics. The positive transformants were verified by a PCR method with the appropriate test primer pairs (Table S30).

### Pathogenicity assays.

Tomato seedlings were grown in the greenhouse at 25°C during 16-h/8-h day/night periods. The V. dahliae wild-type gene deletion mutant and complementation transformant strains were cultured in Czapek Dox liquid medium for 5 days at 25°C. Two-week-old tomato seedlings were inoculated with 1 × 10^7^ conidia/mL V. dahliae strains by a root-dip method for pathogenicity assays as previously described (82, 84) with a few modifications.

Disease symptoms were observed at 3 weeks postinoculation. Fungal biomass in tomato seedlings was determined by qPCR. The V. dahliae ITS sequence was used to quantify fungal colonization (primer pair VdITS1-F/VdSTVe1-R), and the tomato *RuBisCo* gene (primer pair SIRUB-F/R) served as the endogenous plant control (26). All the primer pairs are listed in Table S30. qPCR was performed, and the conditions were used as described above.

### Data availability.

This Whole Genome Shotgun project has been deposited at DDBJ/ENA/GenBank under the accession ACOQX000000000 (GF1192 strain), JACOQY000000000 (GF1300 strain), JACOQZ000000000 (Ud1-4-1 strain), JACORA000000000 (Gf-Cb5 strain), JACORB000000000 (TO22 strain), JACORC000000000 (HoMCLT strain), with the BioProject ID of PRJNA657474, and the Verticilli-Omics database (https://db.cngb.org/Verticilli-Omics/).
