# Supplementary material for: Functional Genomics and Comparative Lineage-Specific Region Analyses Reveal Novel Insights into Race Divergence in Verticillium dahliae
Source: Microbiol Spectr. 2021 Dec 22;9(3):e01118-21. doi: 10.1128/Spectrum.01118-21 (PMC8694104; doi:10.1128/Spectrum.01118-21)
Supplement: SUPPLEMENTAL FILE 2 — Supplemental material. Download SPECTRUM01118-21_Supp_1_seq1.pdf, PDF file, 5.6 MB [file spectrum01118-21_supp_1_seq1.pdf]

Supplementary Material

Supplementary file 1

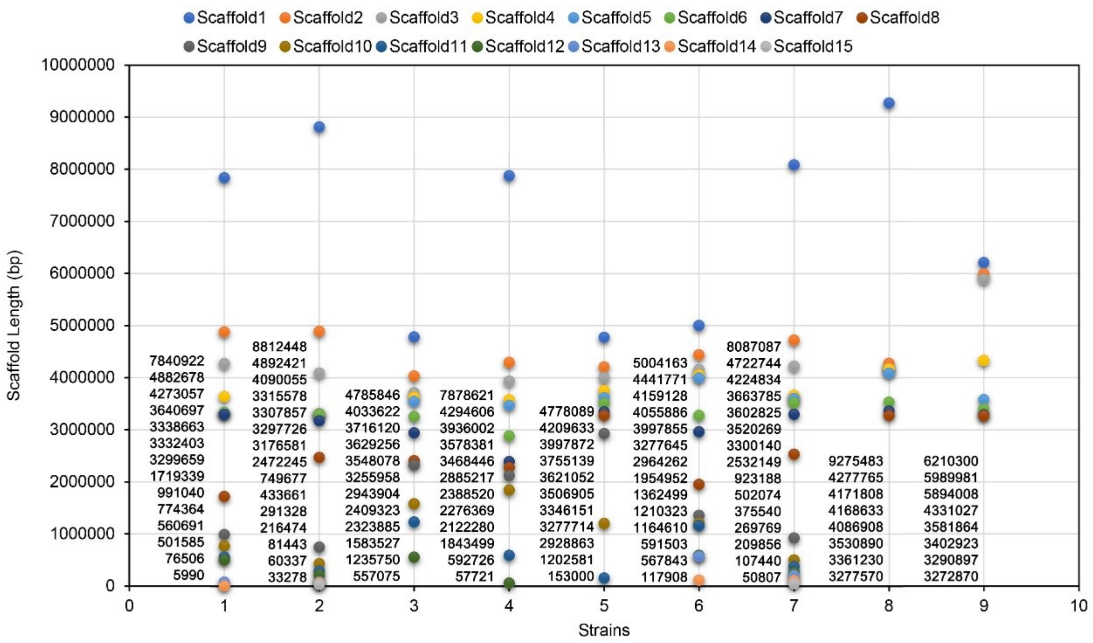

Fig S1 Length information of the assembled genome sequences. JR2 (GenBank assembly accession: GCA_000400815.2) and VdLs.16 (GenBank assembly accession: GCA_013170945.1) are the two released genomes.

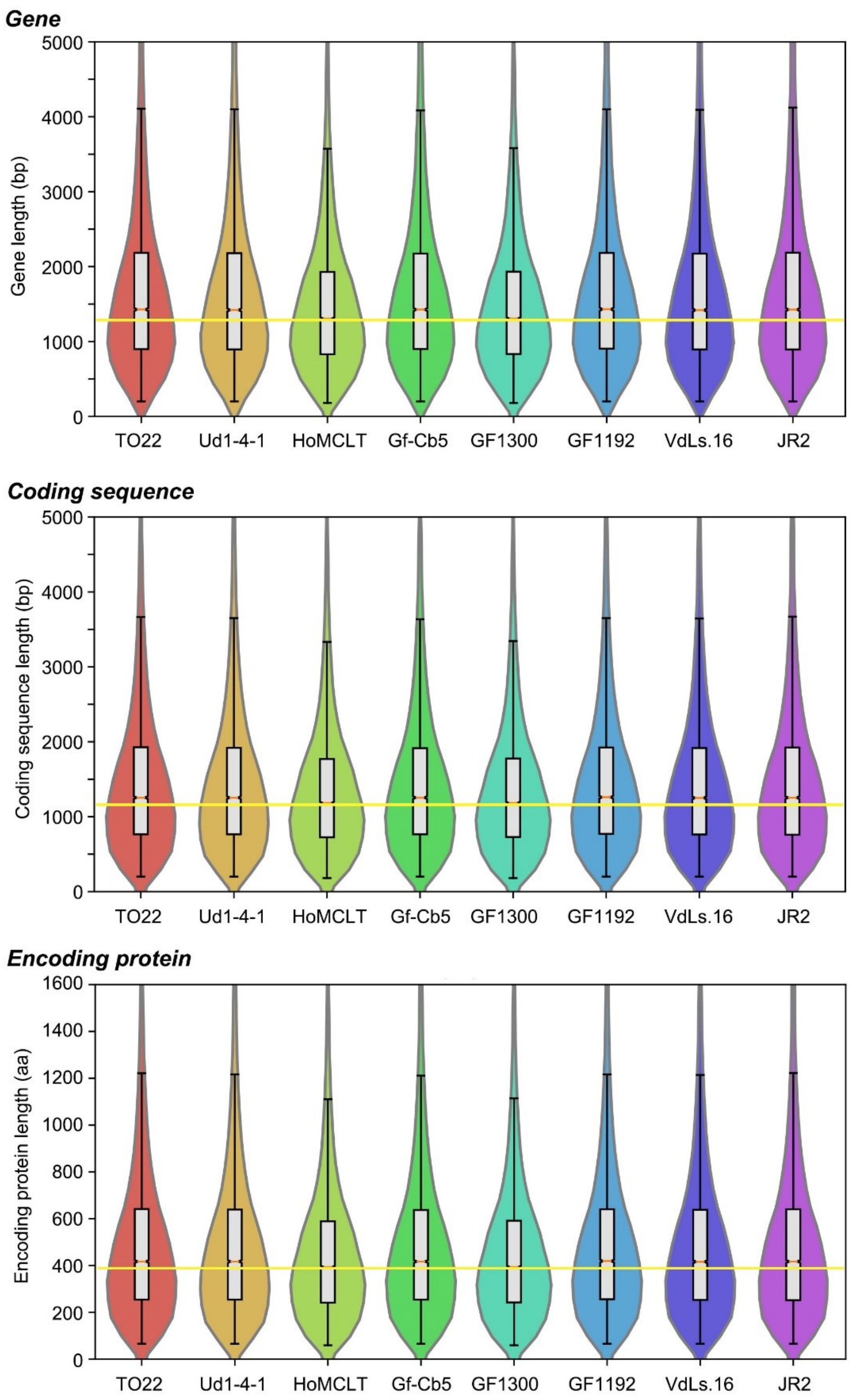

Fig S2 Length information of genes and coding sequences and protein coding sequences among the sequenced genomes. The orange line in the box represents the median value of gene, coding sequence or encoded protein for each genome. The yellow line is the reference that was drawn by the median value derived from the HoMCLT genome.

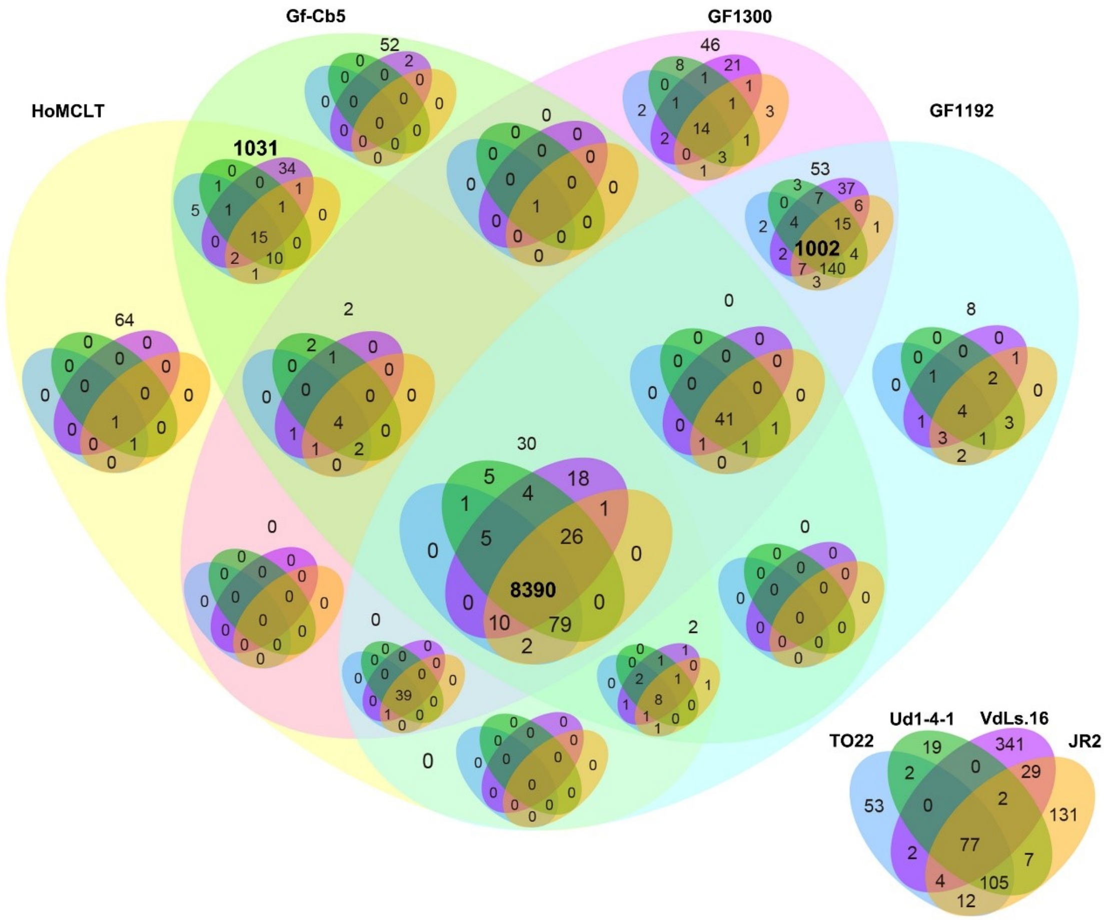

Fig S3 Orthologue clustering among three races in *Verticillium dahliae*. The orthologues were clustered by the OrthMCL with the parameters of coverage and identities >30%.

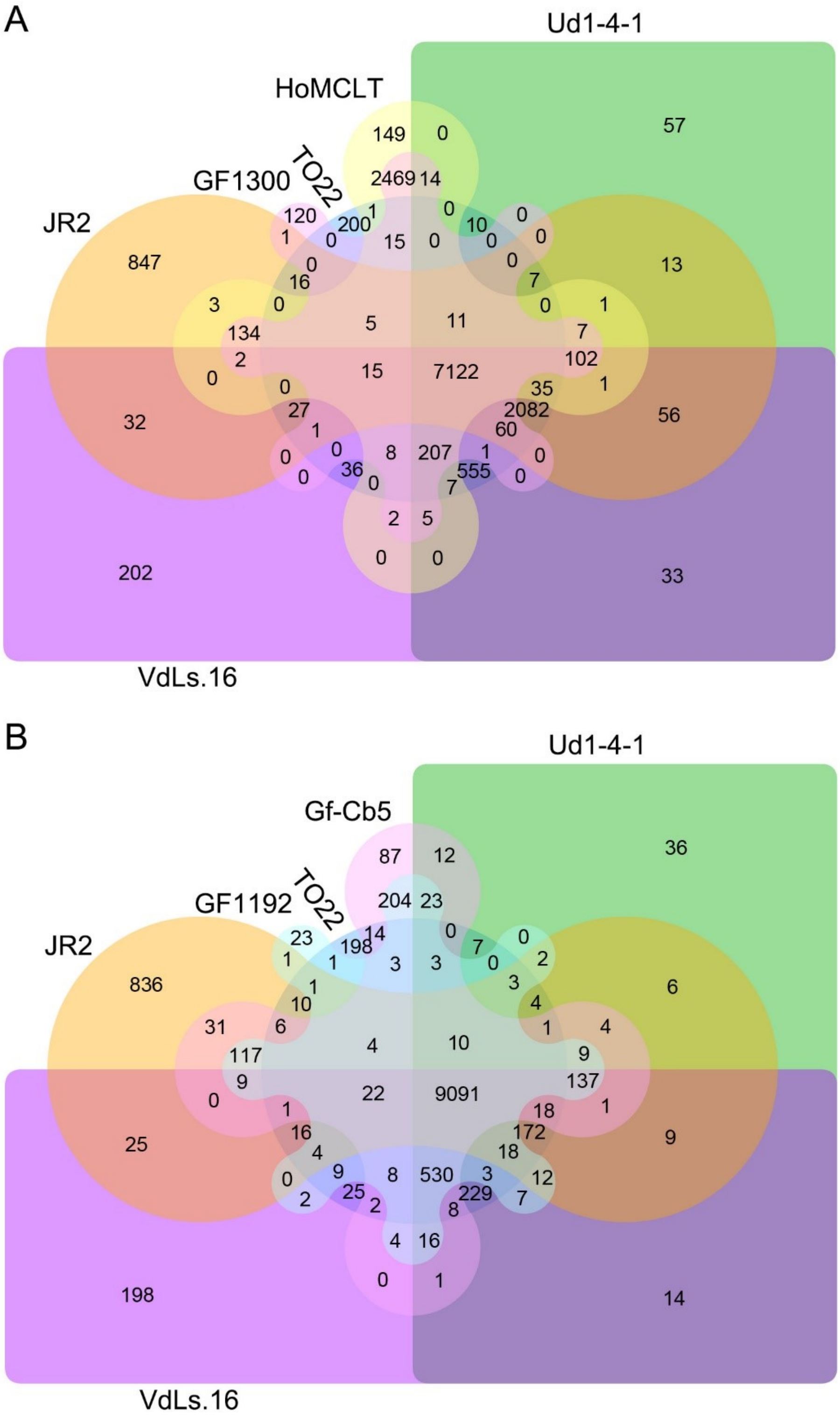

Fig S4 Orthologue clustering of race 3 genomes against race 1 and race 2 in *Verticillium dahliae*. (A) The orthologue relationship of two race 3 strains (HoMCLT and GF1300) with those of race 1 and race 2 genomes. (B) Venn plot of two race 3 strains (Gf-Cb5 and GF1192) with race 1 and race 2 genomes.

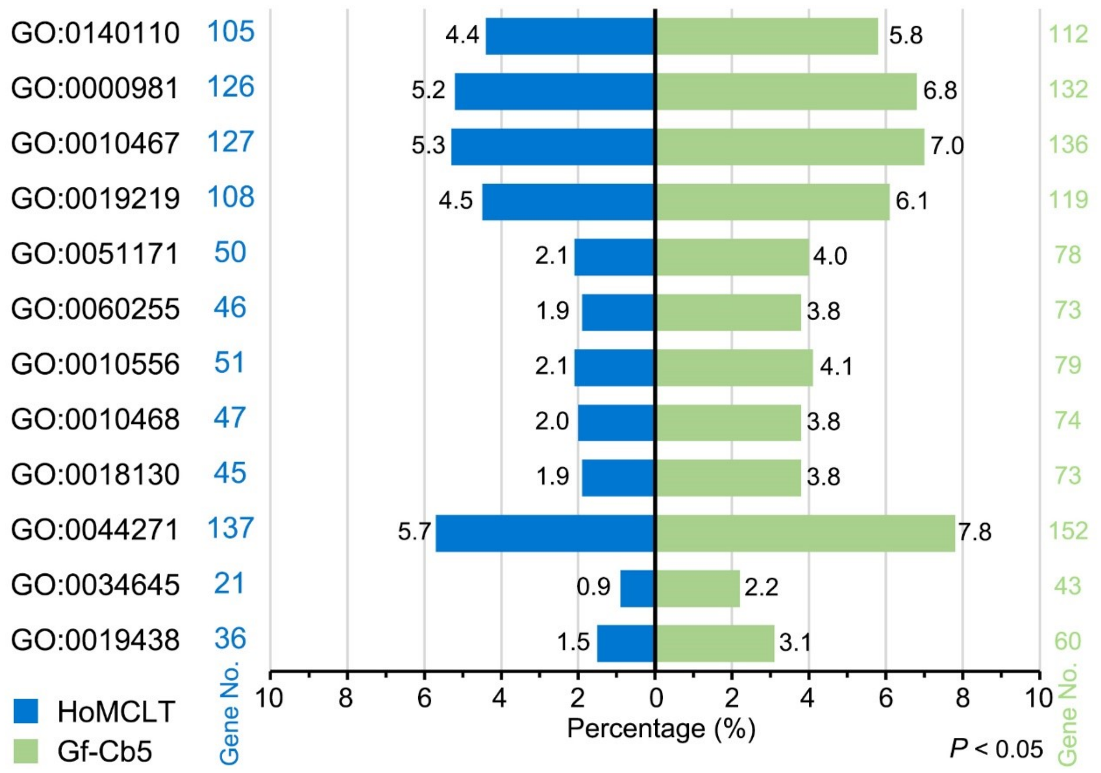

Fig S5 GO annotation of the specific orthologues within the divergent strains of race 3. Two race 3 strains (HoMCLT and GF1300) with 2401 specific orthologues were compared to other the two race 3 strains (Gf-CB5 and GF1192) with 1929 specific orthologues. The specific orthologous proteins encoded by HoMCLT and Gf-Cb5 were collected for GO annotation. The percentage in the X-axis represents the ratio of gene number in each category compared to the total genes from the HoMCLT and Gf-Cb5 genomes. The significant clusters were selected by the *Pearson Chi-Square* test, $P < 0.05$.

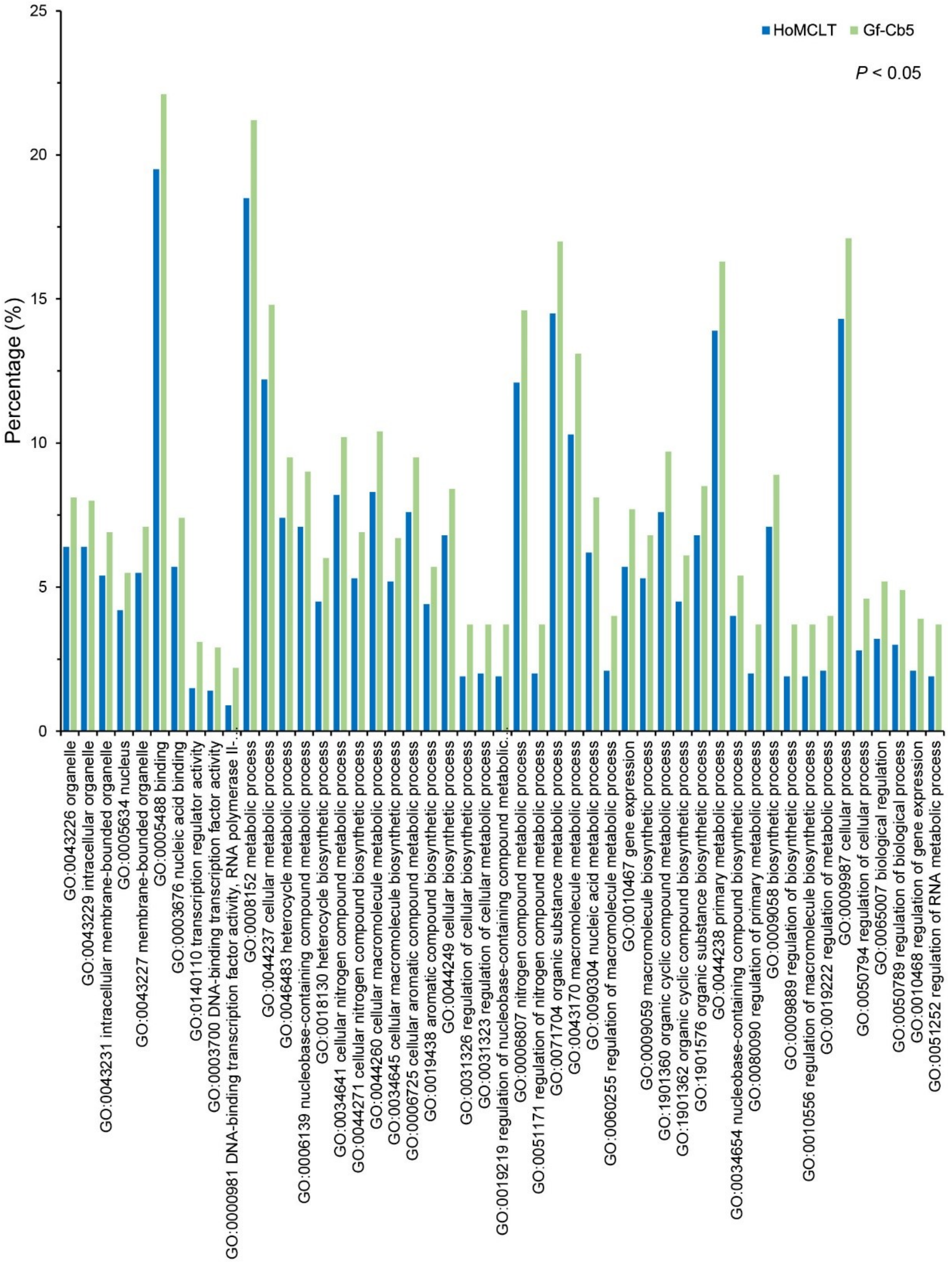

Fig S6 Gene ontology of the divergence orthologue within the race 3 genomes. Two race 3 strains (HoMCLT and GF1300) have 2,401 specific orthologues compared to other two race 3 strains (Gf-CB5 and GF1192) that have 1,929 specific orthologues. The specific orthologue genes encoded by HoMCLT and Gf-Cb5 were collected for GO annotation, and significant clusters were selected by the *Pearson Chi-Square* test, $P < 0.05$.

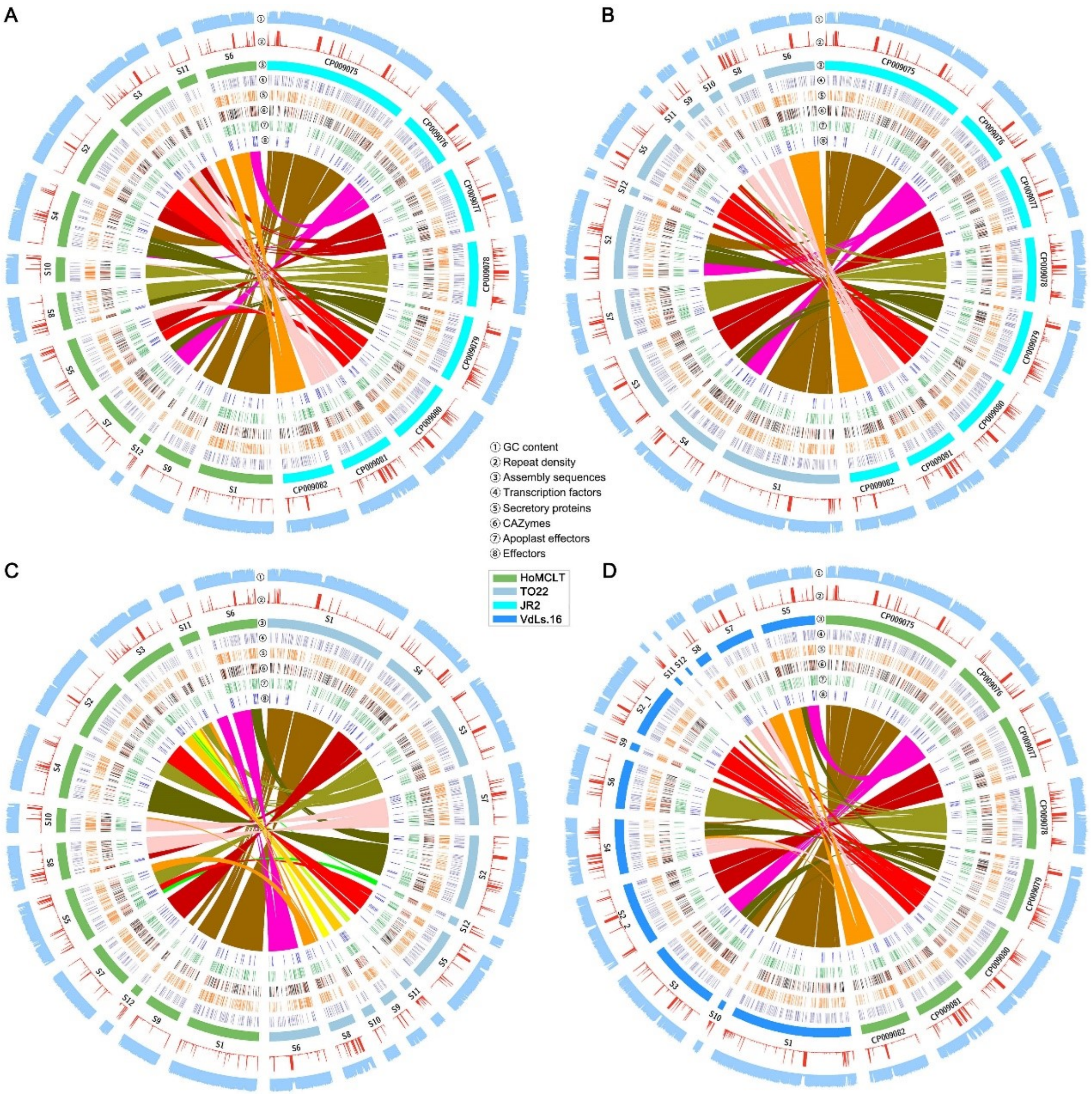

Fig S7 Genome synteny among three races in *Verticillium dahliae*. (A) race 1 strain JR2 vs race 2 strain TO22; (B) race 1 strain JR2 vs race 3 strain HoMCLT; (C) race 3 strain HoMCLT vs race 2 strain TO22. (D) Within race 1 stains, JR2 vs VdLs.16. Synteny between the genomes was constructed by using 5 kb non-overlapping windows.

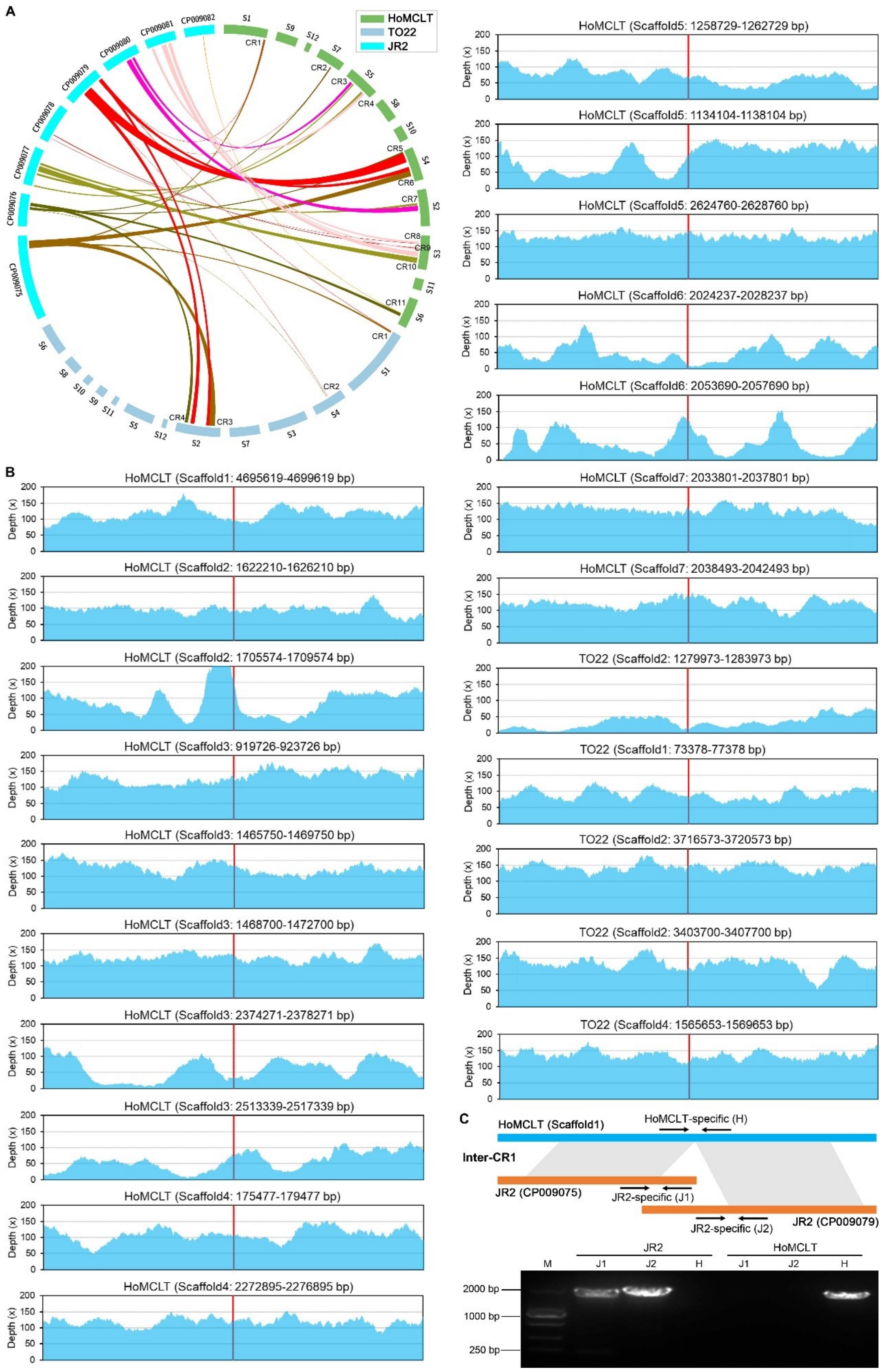

Fig S8 Validating the reliability of the chromosome rearrangement sites by the pair-end reads mapping. (A) Synteny analysis of chromosome rearrangement sites (CRs)

between HoMCLT or TO22 genome versus JR2 genome. (B) The pair-end reads were mapped to the sequence of chromosome rearrangement sites with the flanking sequences (± 2000 bp), the depth for each base was calculated by the coverage read groups and represented graphically. The red line represents the chromosome rearrangement sites in the HoMCLT or TO22 genome versus the JR2 genome. There were only two chromosome rearrangement sites, HoMCLT (Scafflod 8: 589084-593084 bp) and HoMCLT (Scafflod 10: 1433102-1437102 bp), without insufficient support by read mapping. (C) Determination of chromosome rearrangement sites between HoMCLT and JR2 genome. The primer pair (H-CR1-F/R) across the chromosome rearrangement sites in HoMCLT can be amplified from the HoMCLT genomic DNA, but they failed in the JR2 genomic DNA; The primer pairs (J1-CR1-F/R and J2-CR1-F/R) from the synteny blocks of JR2 genome amplified from the JR2 genomic DNA, but failed in the HoMCLT genomic DNA.

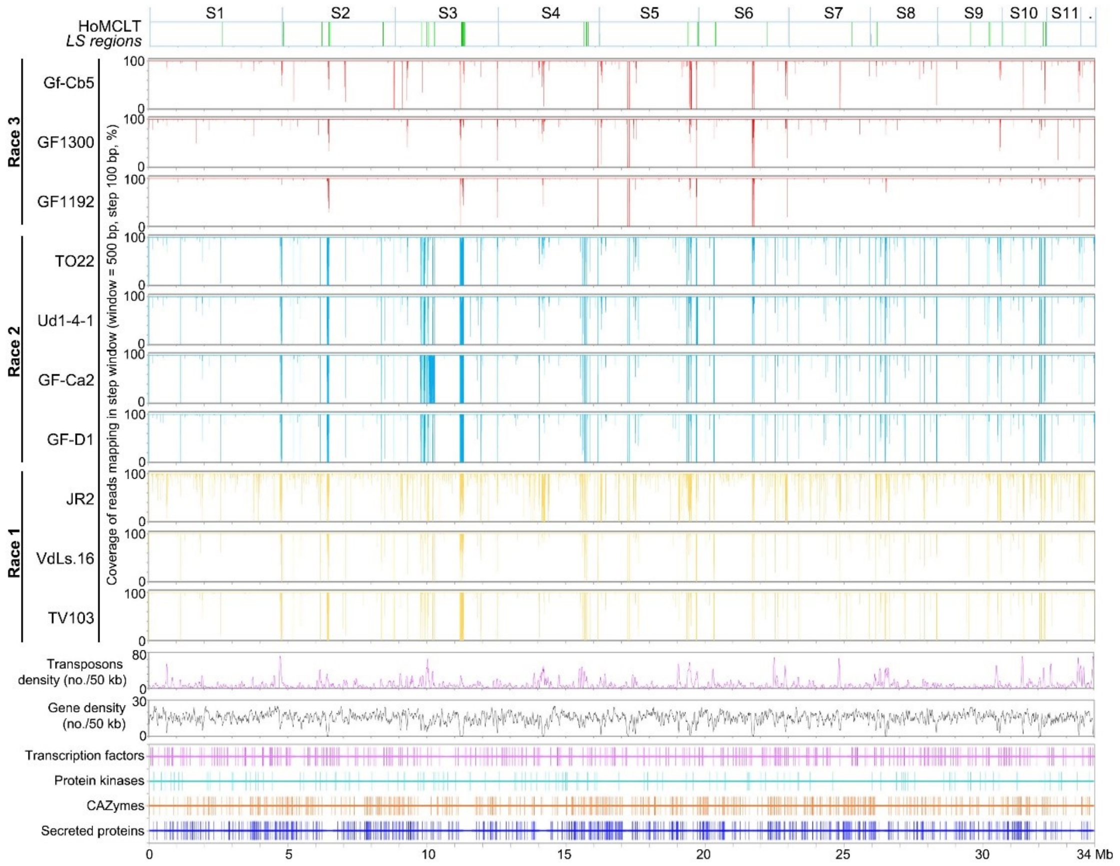

Fig S9 Race 3 lineage-specific regions by coverage for each sequenced strain as compared to the reference genome of HoMCLT. The coverage was calculated by the step windows (window length: 500 bp; step: 100 bp) in each sequenced strain and the reference genomes of JR2 and VdLs.16. Step windows with coverage > 50% and depth > 2 × by race 3 strains, but coverage < 50% in the race 2 and race 1 strains were colored in green, to form the lineage specific regions in the top box. "Sn" represents the assembly Scaffolds of HoMCLT genome. The short reads of JR2 genome were downloaded from the NCBI database (SRA: SRR515981).

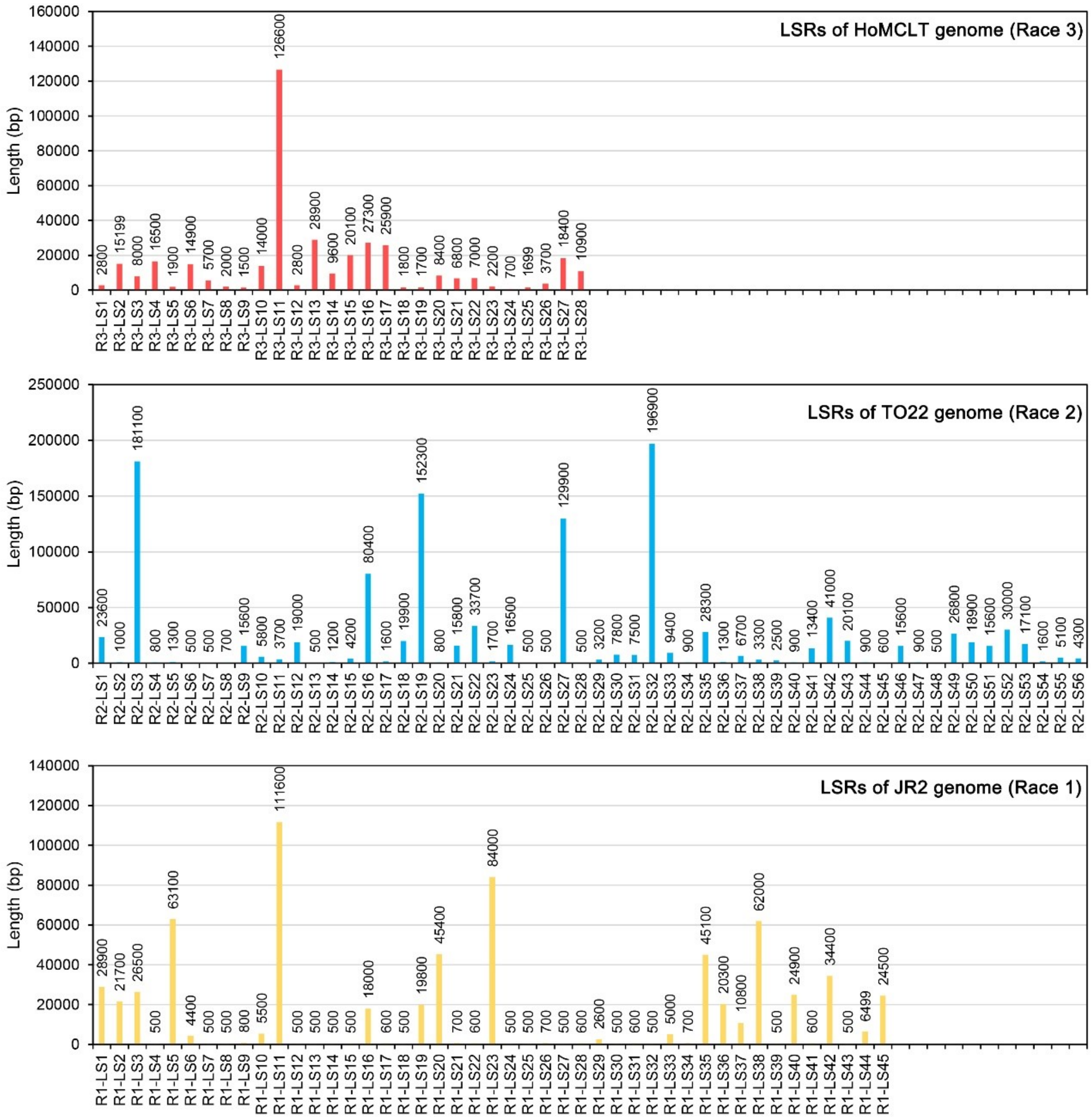

Fig S10 Statistics of the length of LSRs among the HoMCLT, TO22 and JR2 genomes.

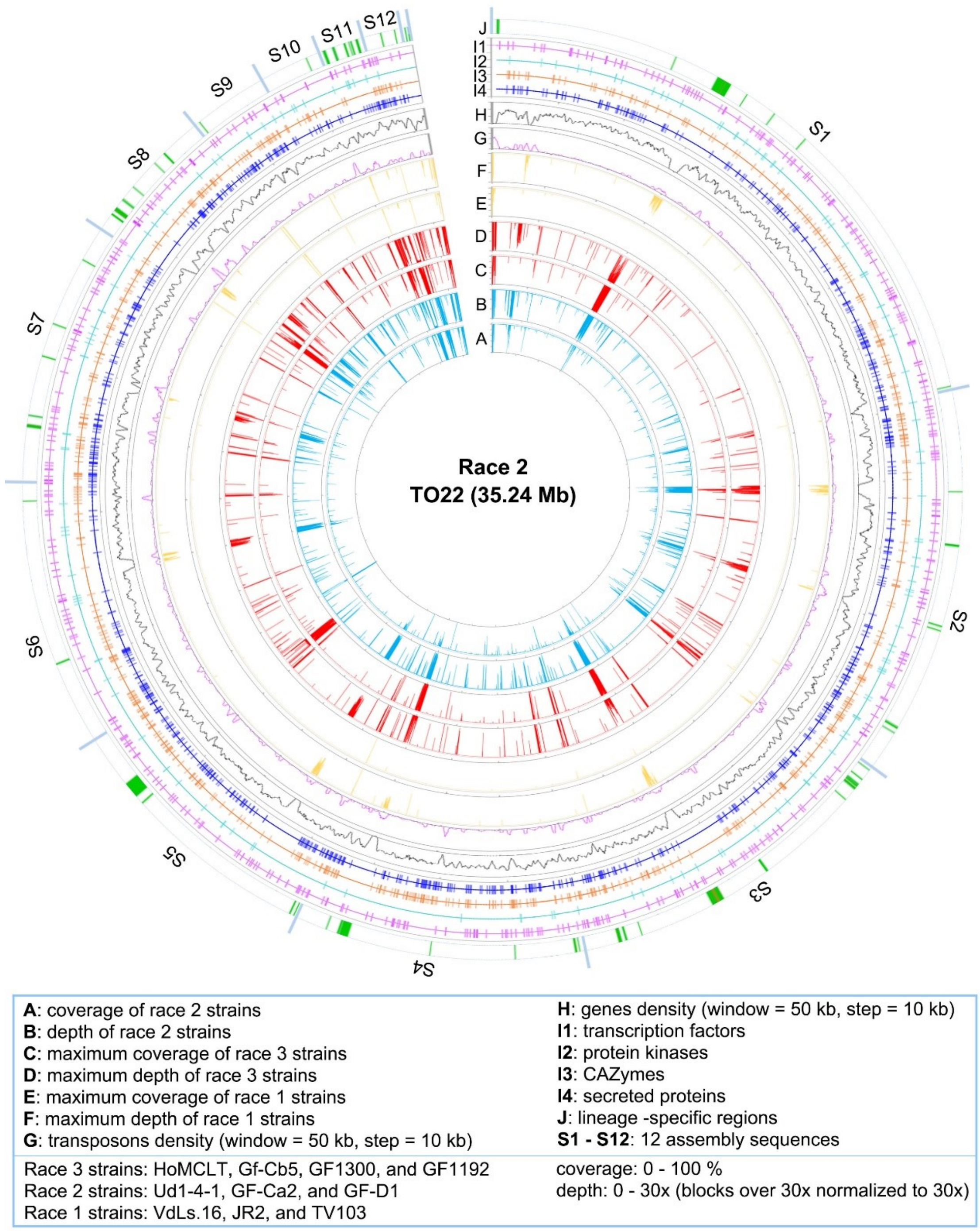

Fig S11 Identification of lineage-specific regions in race 2 relative to race 3 and race 1 in *Verticillium dahliae*. See the legend also in Figure 5.

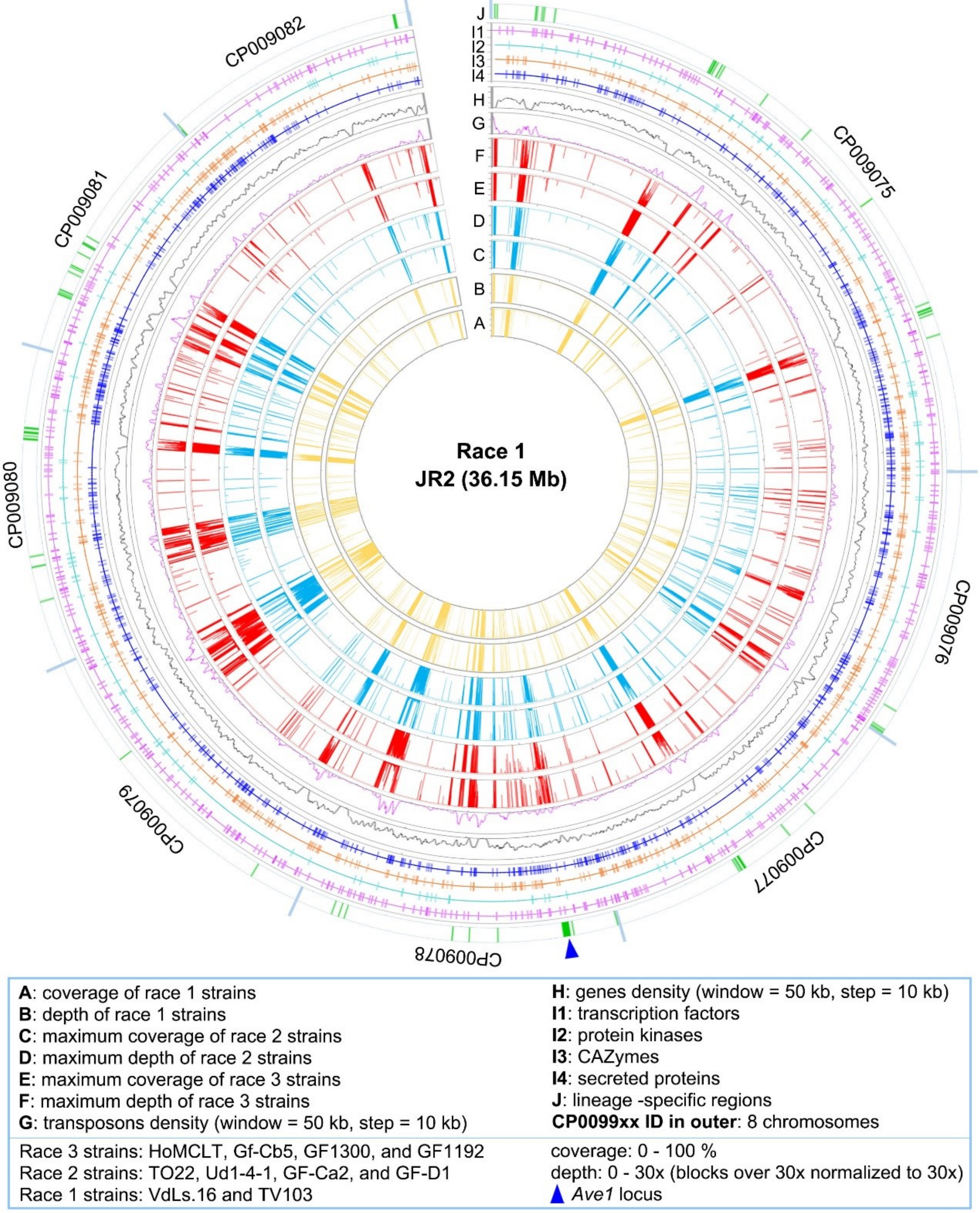

Fig S12 Identification of lineage-specific regions in race 1 relative to race 3 and race 2 in *Verticillium dahliae*. See the legend also in Figure 5.

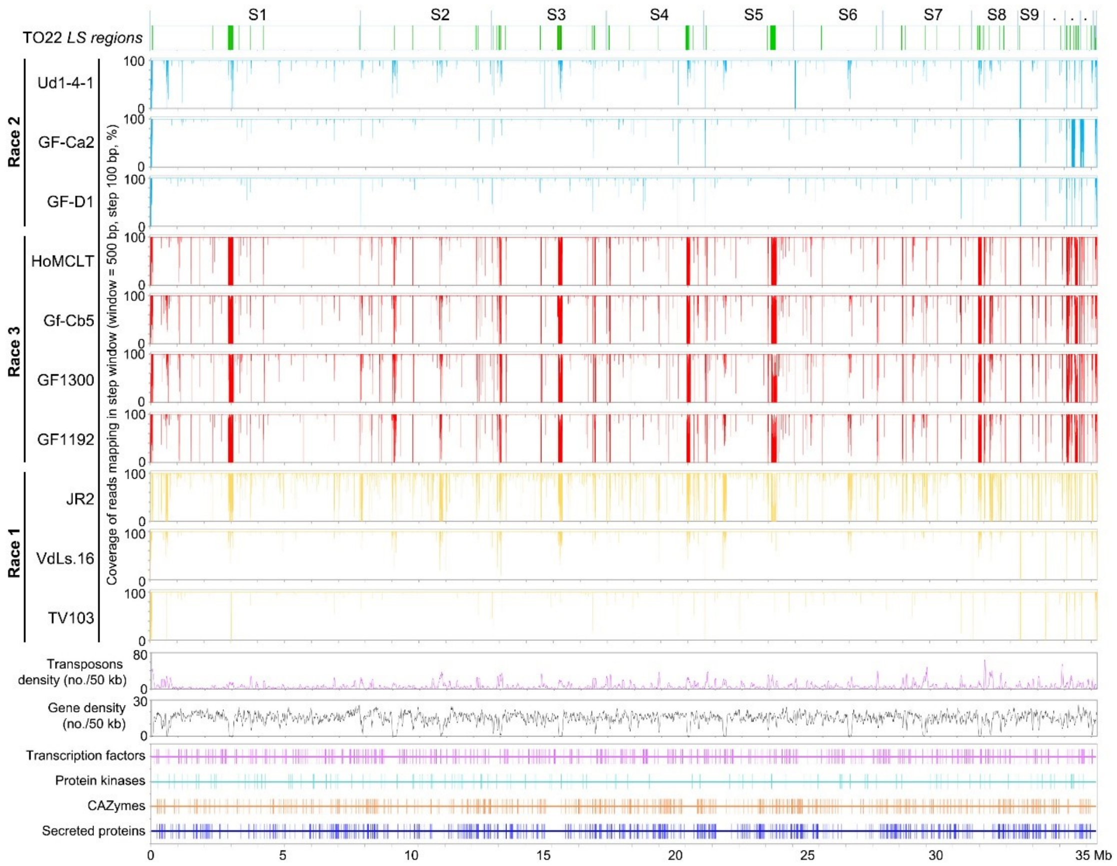

Fig S13 Examination of race 2 lineage-specific regions by coverage for each sequenced strain relative to the reference genome of TO22. See also the legend of Figure S9.

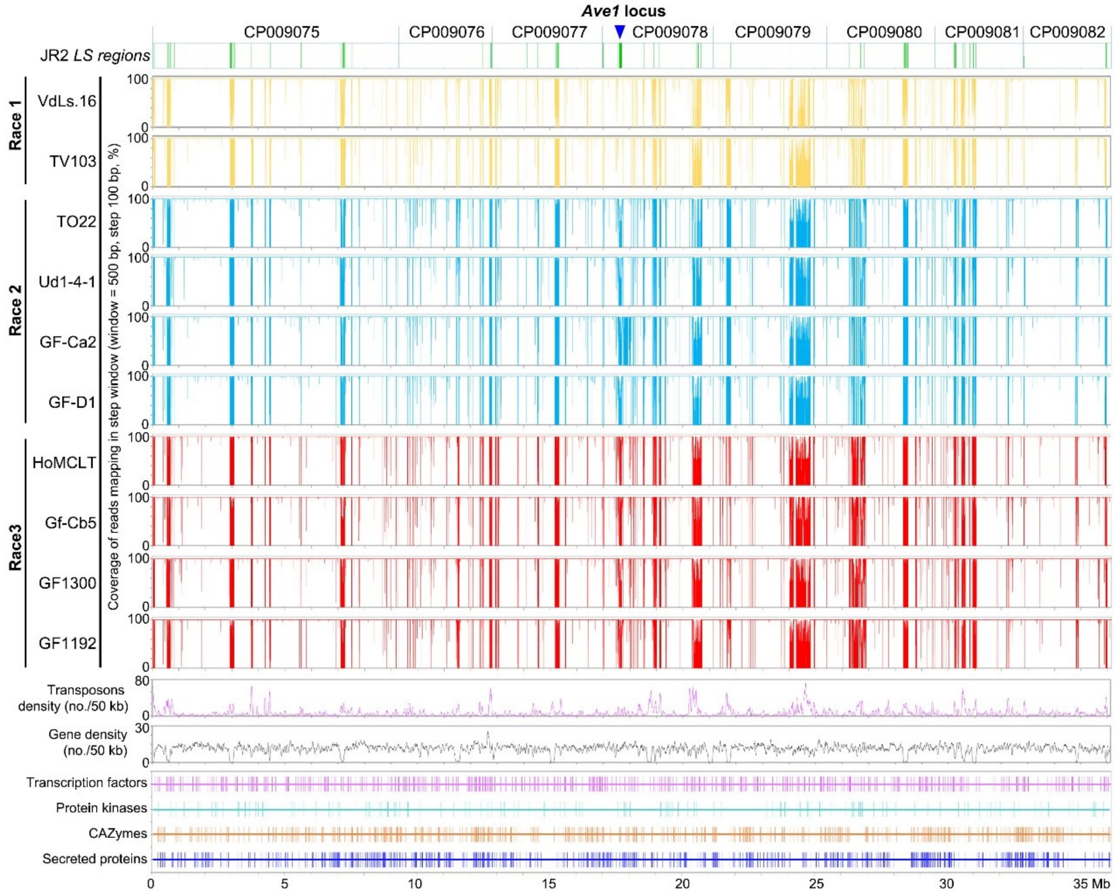

Fig S14 Examination of race 1 lineage-specific genomic regions by coverage for each sequenced strain relative to the reference genome of JR2. See also the legend of Figure S9.

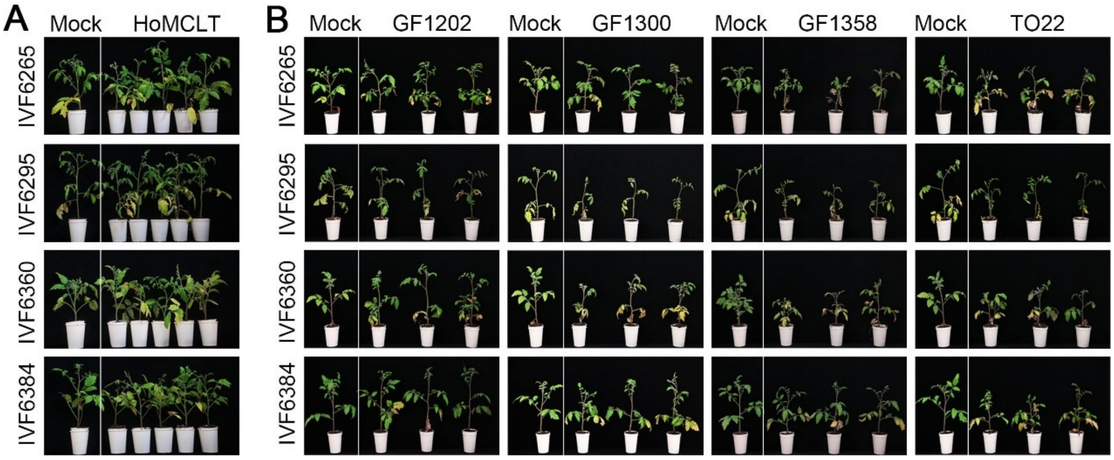

Fig S15 Identification of the resistance in germplasm lines against race 3 strains but susceptible to race 2 and race 1 strains. Three-week-old tomato germplasm seedlings were root-dip inoculated with the *V. dahliae* strains of three races, root treatment with sterile water was set as negative control. Phenotype was photographed at 21 days after inoculation on tomato plants. (A) Tomato germplasms resistant to the race 3 strain HoMCLT. (B) Tomato germplasms susceptible to the race 2 strains.

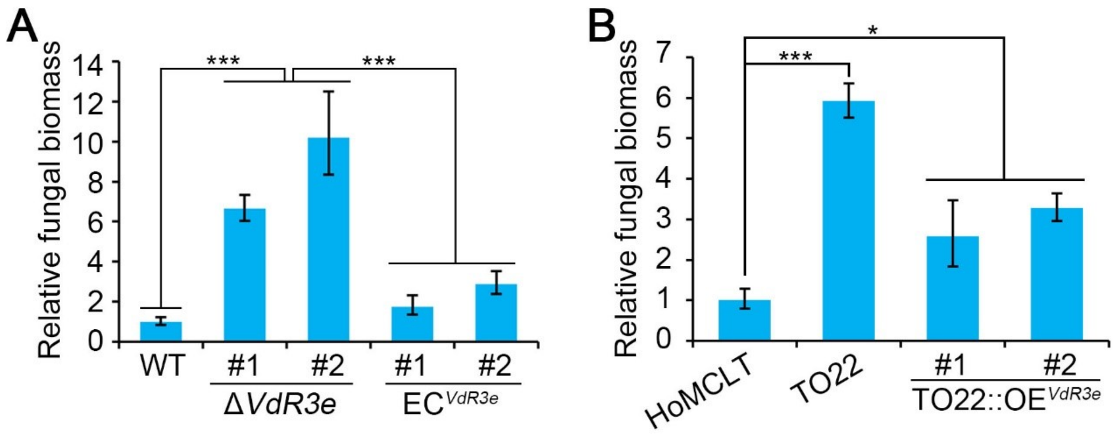

Fig S16 Quantification polymerase chain reaction (qPCR) of fungal biomass of *V. dahliae* strains that carried or lacked (deleted) race 3 candidate avirulence gene (*VdR3e*) that infected resistant tomato germplasm IVF6384. Error bars represent standard errors of the mean. Quantification of the fungal biomass indicated strains in infected tomato plants by qPCR. DNA was amplified from host root tissues using *V. dahliae* ITS sequence primers. (A) Race 3 *VdR3e* deletion and complementary race 3 strain HoMCLT. (B) Overexpressed the *VdR3e* on race 2 strain TO22. Asterisks * and *** indicate significant differences of $P< 0.05$ and $P< 0.001$ respectively; calculated by unpaired Student's *t*-tests.

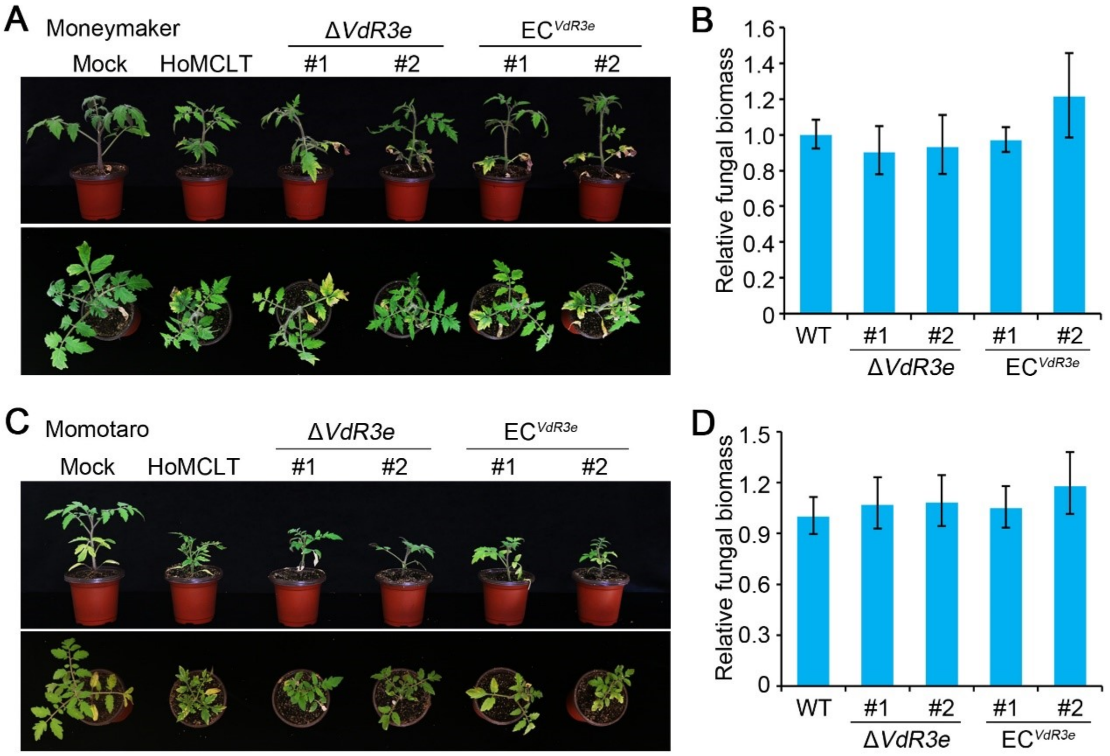

Fig S17 Pathogenicity assay of the *VdR3e* deletion and complementary strains on tomato. Two-week-old tomato seedlings were root-dip inoculated with the two independent *VdR3e* deletion strains and two complementary transformants of re-introducing *VdR3e* with the HoMCLT background, race 3 strain HoMCLT and treatment with sterile water were set as the positive and negative control, respectively. (A, C) Phenotype was photographed at 21 days after inoculation on host plants. (B, D) Quantification the fungal biomass of the indicated strains in infected host plants by qPCR. DNA was amplified from host stem tissues using *V. dahliae* ITS sequence primers. Error bars represent standard error. Statistical significance was calculated by an unpaired Student's *t*-tests. (A, B) cv. Moneymaker, (C, D) cv. Momotaro (1).

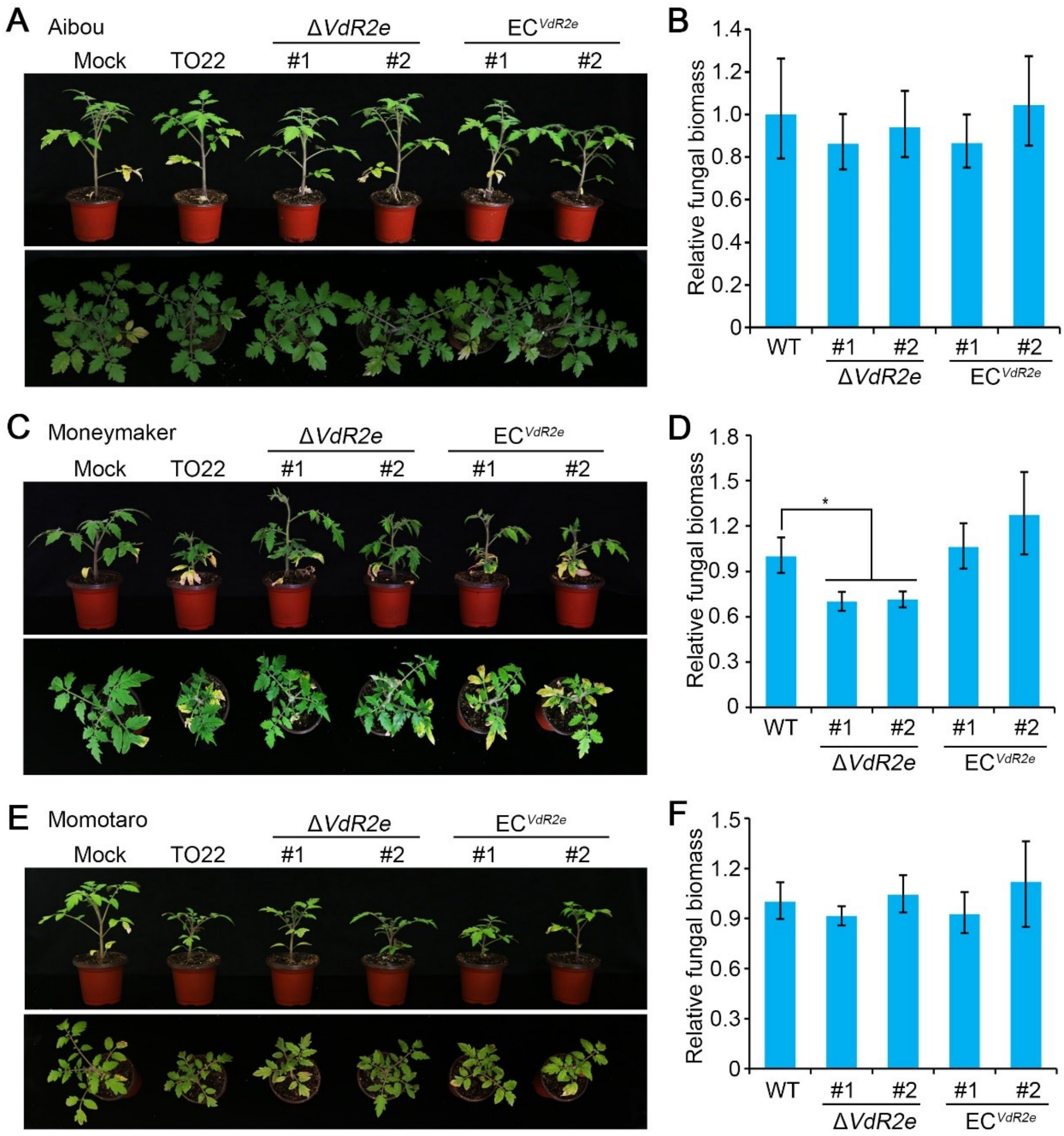

Fig S18 Virulence assay of the *VdR2e* deletion and complementary strains on tomato. Two-week-old tomato seedlings were root-dip inoculated with the two independent *VdR2e* deletion strains and two complementary transformants of re-introducing *VdR2e* with the TO22 background, race 2 strain TO22 and treatment with sterile water were set as the positive and negative control, respectively. (A, C, E) Phenotype was photographed at 21 days after inoculation on tomato plants. (B, D, F) Quantification the fungal biomass of the indicated strains in infected host plants by qPCR. DNA was amplified from host stem tissues using *V. dahliae* ITS sequence primers. Error bars represent standard error. Statistical significance was calculated by an unpaired Student *t*-test. * represent $P < 0.05$. (A, B) cv. Aibou (1), (C, D) cv. Moneymaker, (E, F) cv. Momotaro (1).

Supplementary file 2

Table S1 Non-race 1 strains for genome sequencing in this study (1).

Table S2 Clean reads sequenced by PacBio RS II and Illumina technologies.

Table S3 Information on genome assembly of the sequencing genomes.

Table S4 Length of the assembled sequences among the sequenced genomes.

Table S5 Predicted transposons among the sequenced genomes.

Table S6 Annotated CAZymes among the sequenced genomes.

Table S7 Annotated protein kinase (PKs) subfamily among the sequenced genomes.

Table S8 CAZymes sub-family statistics among the sequenced genomes.

Table S9 Annotated encoding proteins involved in plant cell wall degrading enzymes (CWDEs) among the sequenced genomes.

Table S10 Annotated pathogen-host interaction (PHI) homologs among the sequenced genomes.

Table S11 Annotated transcription factors among the sequenced genomes.

Table S12 Annotated conserved domains encoding proteins among the sequenced genomes.

Table S13 Analysis of the functional divergence among the sequenced genome by the conserved domain annotation with the InterPro database.

Table S14 The specific orthologues among races in *Verticillium dahliae.*

Table S15 Statistics on the pathogenicity-related genes in the specific orthologues among the three races in *Verticillium dahliae.*

Table S16 Orthologues statistics and genes among the sequenced genomes.

Table S17 Comparative synteny analysis of the eight chromosome centromere regions between the JR2 and HoMCLT/TO22 genomes (2).

Table S18 Information on the inter-chromosomal rearrangements among the three races in *Verticillium dahliae.*

Table S19 The number of pathogenicity-related genes among the eight chromosomes in *Verticillium dahliae.*

Table S20 Information on the LS regions among the races in *Verticillium dahliae* genome.

Table S21 Prediction and annotation of the LS regions among the races of *Verticillium dahliae* genome.

Table S22 Number of potential pathogenicity related genes in LSRs among the three races in *Verticillium dahliae.*

Table S23 Comparison of the conserved domains of the encoded genes in LSRs among the three races in *Verticillium dahliae* (3, 4, 5).

Table S24 Information on the gene prediction and annotation of the LSRs in *Verticillium dahliae* race 1 strain JR2.

Table S25 Information on the gene prediction and annotation of the LSRs in *Verticillium dahliae* race 2 strain TO22.

Table S26 Information on the gene prediction and annotation of the LSRs in *Verticillium dahliae* race 3 strain HoMCLT.

Table S27 Identification of race-specific genes from the encoded genes in LSRs among the three races in *Verticillium dahliae.*

Table S28 Detection of the races in *Verticillium dahliae* population (1).

Table S29 Resistance and susceptibility of tomato germplasm lines against *Verticillium dahliae* strains from the three races.

Table S30 Primers used in this study (1).

References

1. Usami T, Momma N, Kikuchi S, Watanabe H, Hayashi A, Mizukawa M, Yoshino K, Ohmori Y. 2017. Race 2 of *Verticillium dahliae* infecting tomato in Japan can be split into two races with differential pathogenicity on resistant rootstocks. Plant Pathol 66:230–238. https://doi.org/10.1111/ppa.12576.

2. Seidl MF, Kramer HM, Cook DE, Fiorin GL, van den Berg GC, Faino L, Thomma BP. 2020. Repetitive elements contribute to the diversity and evolution of centromeres in the fungal genus *Verticillium*. mBio 11:e01714-1720. https://doi.org/10.1128/mBio.01714-20.

3. Horton P, Park K, Obayashi T, Fujita N, Harada H, Adams-Collier CJ, Kenta N. 2007. WoLF PSORT: protein localization predictor. Nucleic Acid Res 35:W585–587. https://doi.org/10.1093/nar/gkm259.

4. Käll L, Krogh A, Sonnhammer EL. 2004. A combined transmembrane topology and signal peptide prediction method. J Mol Biol 338:1027–1036. https://doi.org/10.1016/j.jmb.2004.03.016.

5. Krogh A, Larsson B, von Heijne G, Sonnhammer EL. 2001. Predicting transmembrane protein topology with a hidden Markov model: application to complete genomes. J Mol Biol 305:567–580. https://doi.org/10.1006/jmbi.2000.4315.
